# Structural and mechanistic basis for preferential deadenylation of U6 snRNA by Usb1

**DOI:** 10.1093/nar/gky812

**Published:** 2018-09-12

**Authors:** Yuichiro Nomura, Daniel Roston, Eric J Montemayor, Qiang Cui, Samuel E Butcher

**Affiliations:** 1 Department of Biochemistry, University of Wisconsin, Madison, WI 53706, USA; 2 Department of Chemistry, University of Wisconsin, Madison, WI 53706, USA; 3 Department of Biomolecular Chemistry, University of Wisconsin, Madison, WI 53706, USA

## Abstract

Post-transcriptional modification of snRNA is central to spliceosome function. Usb1 is an exoribonuclease that shortens the oligo-uridine tail of U6 snRNA, resulting in a terminal 2′,3′ cyclic phosphate group in most eukaryotes, including humans. Loss of function mutations in human Usb1 cause the rare disorder poikiloderma with neutropenia (PN), and result in U6 snRNAs with elongated 3′ ends that are aberrantly adenylated. Here, we show that human Usb1 removes 3′ adenosines with 20-fold greater efficiency than uridines, which explains the presence of adenylated U6 snRNAs in cells lacking Usb1. We determined three high-resolution co-crystal structures of Usb1: wild-type Usb1 bound to the substrate analog adenosine 5′-monophosphate, and an inactive mutant bound to RNAs with a 3′ terminal adenosine and uridine. These structures, along with QM/MM MD simulations of the catalytic mechanism, illuminate the molecular basis for preferential deadenylation of U6 snRNA. The extent of Usb1 processing is influenced by the secondary structure of U6 snRNA.

## INTRODUCTION

**Figure 1.**
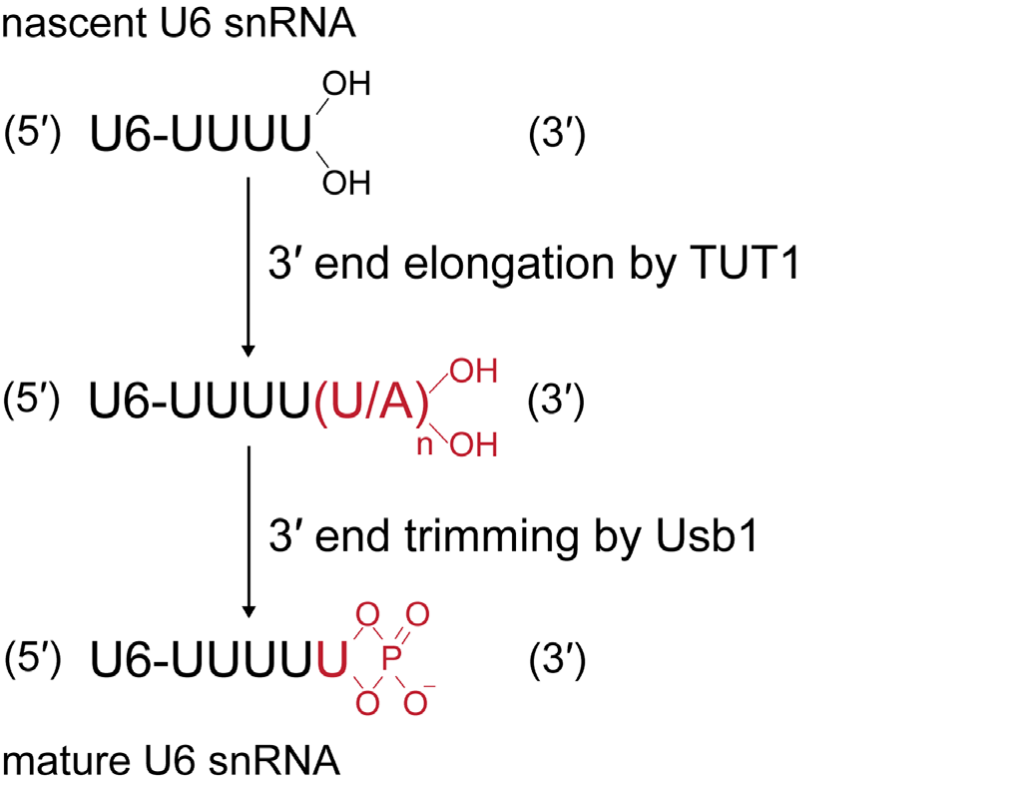
Overview of 3′-end processing of U6 snRNA. Termination of transcription by RNA polymerase III leaves a polyuridine tract at the 3′ end of U6 with heterogeneous length. Subsequent elongation by TUT1 adds additional uridine and adenosine nucleotides, which is counteracted by the 3′ exoribonuclease Usb1. *In vivo*, the dominant form of U6 has five uridines and a terminal 2′,3′-cyclic phosphate moiety that results from opposing 3′-end elongation and trimming.

Precursor messenger RNA contains intervening sequences, or introns, that must be removed by the spliceosome, a large and highly dynamic macromolecular complex that contains over a hundred proteins and five small nuclear RNAs (U1, U2, U4, U5 and U6 snRNAs) (1). In addition, humans and other organisms also have a minor spliceosome that is highly similar to the major spliceosome but has U11, U12, U4atac and U6atac snRNAs instead of the U1, U2, U4 and U6 snRNAs, respectively (2). During spliceosome assembly, individual snRNAs are recruited to spliceosomes in the form of small nuclear ribonucleoproteins (snRNPs). In humans, the maturation pathway of U6 and U6atac snRNAs and their incorporation into snRNPs is distinct from the other snRNAs (Figure 1). For example, U6 and U6atac snRNAs are transcribed by RNA polymerase III and subsequent maturation occurs in the nucleus (3,4,5,6,7). In contrast, all other snRNAs are synthesized by RNA polymerase II, transported into the cytoplasm for post-transcriptional processing and are bound by Sm proteins before re-entry into the nucleus (8).

Nascent human U6 and U6atac snRNA transcripts have heterogeneous polyuridine 3′ ends, owing to the stochastic nature of RNA polymerase III termination (3,9). The nascent U6 and U6atac transcripts terminate with 2′,3′ *cis* diols (10) and are initially bound by La protein (11,12) before post-transcriptional 3′ end extension by TUT1 (4), and shortening by Usb1 (6,7,13). The resultant U6 snRNA species have predominantly five uridine nucleotides with a 2′,3′-cyclic phosphate that facilitates binding of Lsm2–8 instead of La protein (Figure 1) (14,15).

TUT1 catalyzes 3′ polyuridylation of U6 and U6atac snRNAs (16), while Usb1 has the opposing activity of removing 3′ uridines from U6 (Figure 1). Loss of function mutations in the human *USB1* gene result in the rare genetic disorder poikiloderma with neutropenia (PN) (17,18,19). In cells derived from PN patients, U6 and U6atac snRNAs are abnormally elongated with more U’s and are frequently monoadenylated at their 3′ ends, or less frequently have two or three terminal adenosines (13,20). The same results are observed in *Schizosaccharomyces pombe* cells lacking Usb1 (also called Mpn1) (20). These aberrant U6 3′ tails are produced by an unidentified polymerase. The polymerase responsible for adenylation is unlikely to be the TRAMP-associated adenylase Trf4, since deletion of the *S. pombe* ortholog Cid14 still results in adenylated U6 when Usb1 is also deleted (20). Possibly, TUT1 (also known as Star-PAP) may be the responsible adenylase, as it catalyzes 3′ polyadenylation of certain mRNAs (21,22,23,24,25). The molecular basis for 3′ adenylation of U6 snRNAs in cells lacking Usb1 has thus far remained unexplained.

Usb1 is a member of the 2H phosphodiesterase superfamily (26), a large enzyme family containing members with known 2′,3′-cyclic or 1″,2″-cyclic phosphodiesterase (CPDase) activity, or 3′-5′ or 2′-5′ phosphodiesterase activity, or 2′,5′-RNA ligase activity (7,27,28,29,30,31,32). The active sites of 2H phosphodiesterase enzymes are characterized by a symmetric shape with two HxT/S motifs centered in a catalytic cleft. Recently determined crystal structures of Usb1 were found to be similar to other 2H phosphodiesterases (13,15) and catalytic mechanisms for Usb1 have been proposed (6,13,15). However, the catalytic mechanism has not been experimentally determined, in part due to a lack of a substrate bound complex with a phosphodiester linkage in the active site.

Here, we use *in vitro* activity assays and kinetic measurements combined with X-ray crystallography and computational simulations to define the catalytic mechanism of Usb1. We show that Usb1 preferentially catalyzes deadenylation over deuridylation of 3′ termini of U6 snRNA. We report three high-resolution co-crystal structures of Usb1 complexed with RNA, which show the structural basis for this catalytic preference and unambiguously identify His120 and His208 as catalytic base and acid, respectively. We leveraged this structural information to simulate the enzymatic mechanism using QM/MM simulations. Together, these data reveal the molecular basis for preferential deadenylation of U6 and U6atac snRNAs by Usb1. Additionally, we show that secondary structure in U6 snRNA has a pronounced effect on how many nucleotides Usb1 can efficiently remove from the 3′ end of U6 snRNA.

## MATERIALS AND METHODS

### Overexpression and purification of human Usb1

The coding sequence of full-length human Usb1 (residues 1–265) was codon optimized for expression in *Escherichia coli*, and synthesized by Integrated DNA Technologies. The DNA fragment was inserted between the NdeI and BamHI sites of pET3a carrying the coding sequence of an octahistidine tag, maltose binding protein and TEV protease cleavage site upstream of the NdeI site. The coding sequence of N-terminally truncated human Usb1 (residues 79–265) was amplified by inverse polymerase chain reaction (PCR) (33) using the plasmid containing full-length Usb1. Mutants of these plasmids were introduced by inverse PCR with primers designed for the target DNA sequence. All PCR products were treated with DpnI, ligated using T4 polynucleotide kinase and T4 DNA ligase (New England Biolabs). The synthesized DNA sequences used in this study are summarized in Supplementary Data. *Escherichia coli* BL21(DE3)pLysS competent cells (Invitrogen) were used for overexpression of recombinant proteins. Transformants were grown at 37°C for 2 days in terrific broth medium supplemented with 1% glycerol, 100 μg/ml of ampicillin and chloramphenicol, and induction of protein synthesis was achieved by the ‘leaky’ nature of the T7 RNA polymerase promoter in plasmid pET3a. Cells were harvested by centrifugation at 4000 *g* for 15 min. The pellet was resuspended in immobilized metal affinity chromatography (IMAC) buffer (50 mM HEPES (pH 7.4), 500 mM NaCl, 25 mM imidazole, 1 mM TCEP-HCl and 10% glycerol) supplemented with DNase I, Lysozyme and protease inhibitors (EMD Millipore), and then disrupted by sonication. The supernatant obtained by centrifugation at 48 000 *g* for 20 min was loaded onto 3 ml of a Ni-NTA agarose resin (QIAGEN) and eluted with IMAC buffer containing 500 mM imidazole. The elute was dialyzed against IEX buffer (20 mM HEPES (pH 7.4), 100 mM NaCl, 1 mM TCEP-HCl and 10% glycerol) supplemented with TEV protease at 4°C overnight. To remove maltose binding protein, the dialysate of full-length Usb1 was purified using an amylose resin (NEB). The resultant fraction was directly applied onto HiTrap Heparin column (GE Healthcare) and eluted with a linear gradient from 100 to 600 mM NaCl in IEX buffer. The dialysate of truncated Usb1 was purified with HiTrap SP column (GE Healthcare) with a linear gradient from 100 to 600 mM NaCl in IEX buffer. The purified proteins used for biochemical assays were stored at −80°C. For crystallization, the final product was further dialyzed against crystallization buffer (5 mM Tris (pH 8.2), 100 mM NaCl and 0.1 mM TCEP-HCl), and stored at −80°C.

### Crystallization and structure determination

Crystals of truncated H208Q-Usb1 bound to RNA oligonucleotides 5′-UUUU-3′ were obtained by hanging drop vapor diffusion with 2 μl of 5 mg/ml protein, 2 μl of 740 μM RNA oligonucleotides (GE Healthcare) dissolved in 100 mM bis-tris (pH 6.5) and 2 μl reservoir solution (100 mM Tris (pH 7.0), 25% SOKALAN CP 42 (Molecular Dimensions) and 10% tetrahydrofuran (Molecular Dimensions)) at 4°C for a week. The crystals were transferred into perfluoropolyether cryo oil (Hampton Research), and flash cooled in liquid nitrogen. The crystals of H208Q-Usb1 bound to RNA oligonucleotides 5′-UUUA-3′ were obtained by hanging drop vapor diffusion with 2 μl of 10 mg/ml protein, 2 μl of 1.3 mM RNA oligonucleotides (GE Healthcare) dissolved in 100 mM bis-tris (pH 6.5) and 2 μl of reservoir solution (100 mM bis-tris (pH 6.3), 25% SOKALAN CP 42 and 1.5% tetrahydrofuran) at 16°C for 7–10 days. The crystals were transferred into a solution (100 mM bis-tris (pH 5.5), 20% PEG 20 000, 20% glycerol and 3 mM RNA oligonucleotides), and allowed to incubate at 16°C overnight. The resultant crystals were harvested and flash cooled in liquid nitrogen. The crystals of truncated wild-type Usb1 with AMP were grown by hanging drop vapor diffusion with 2 μl of 13 mg/ml protein, 2 μl of 25 mM AMP dissolved in 100 mM bis-tris (pH 6.5) and 2 μl of reservoir solution (100 mM bis-tris (pH 6.5), 25% SOKALAN CP 42 and 0.9% tetrahydrofuran) at 16°C for 7–10 days. The crystals were transferred into a solution (100 mM bis-tris (pH 6.5), 20% PEG 20,000 and 20% glycerol), and flash cooled in liquid nitrogen.

Diffraction data were collected at 100 K on beamlines 21-ID-G or 24-ID-C at the Advanced Photon Source. Data were integrated, indexed and scaled with XDS (34) and AIMLESS (35). Initial phases were determined by molecular replacement using Phaser (36) with PDB ID: 5V1M (15), and refinement accomplished by the iterative process of manual model building in Coot (37) and automated refinement in PHENIX (38,39) and Refmac (40,41). All figures were generated with PyMOL (http://www.pymol.org/).

### Preparation of RNA substrates

RNA substrates labeled with 6-carboxyfluorescein at the 5′ end (5′-FAM) and modified with a 2′-deoxyuridine (dU) were purchased from Integrated DNA Technologies. These RNA substrates were purified via 20% polyacrylamide gel containing 8 M urea, followed by further purification with HiTrap Q column (GE healthcare). *In vitro* transcription was performed to synthesize RNA substrates corresponding to human U6 nucleotides 1–107 (U6 1–107) and 1–107 with four additional uridine nucleotides at 3′ end (U6 1–107+4U) (42). DNA sequences corresponding to each RNA were inserted into the pUC57 plasmid containing the T7 promoter with an additional guanine nucleotide at the beginning of the transcript and the HDV ribozyme at the end of the transcript, followed by the BamHI site. The plasmid linearized with the BamHI was used as a template for recombinant His-tagged T7 RNA polymerase. The target RNAs were purified via 10% polyacrylamide gel containing 8 M urea, followed by further purification with HiTrap Q column.

5′-terminally cyanine (5′-Cy5) labeled U6 1–107 and U6 1–107+4U were produced by splinted ligation as previously described (43). The transcripts corresponding to U6 12–107 and U6 12–107+4U were synthesized via *in vitro* transcription as described above. 5′-Cy5 labeled U6 1–11 and the splinted DNA oligonucleotides were purchased from Integrated DNA Technologies.

### Exoribonuclease assays

Exoribonuclease assays were generally carried out at 37°C for 60 min in Usb1 buffer (20 mM HEPES (pH 7.5), 100 mM NaCl, 1 mM ethylenediaminetetraacetic acid ( EDTA) and 1 mM TCEP-HCl), conditions that were found to maximize solubility of the enzyme and are close to the pH optimum (Supplementary Figure S1). Reactions typically contained 1 μM RNA substrate and 0–10 μM Usb1. To determine the length of RNA products processed by Usb1, a marker RNA (^–^OH) was generated by partial hydrolysis at 95°C for 10 min in 50 mM sodium carbonate (pH 9.2) and 1 mM EDTA. The products were analyzed by 20% polyacrylamide gel containing 8 M urea. The intensity of each RNA product was visualized by FAM fluorescence on a Typhoon FLA 9000 (GE Healthcare).

For determination of kinetic parameters of Usb1 for U6 101–106 with a 2′-deoxyuridine at position 106 and two additional 3′ adenosine nucleotides (U6 101–106+AA), assays were performed in Usb1 buffer containing 0–55 μM substrate using 2-fold dilutions (0.85, 1.7, 3.4, 6.8, 13.7, 27.5 and 55 μM) and 0.1 μM Usb1 at 37°C for 10 min. For U6 101–106+UA, assays were performed in Usb1 buffer containing 0–40 μM substrate using 2-fold dilutions (0.62, 1.25, 2.5, 5, 10, 20 and 40 μM) and 0.1 μM Usb1 at 37°C for 5 min. For U6 101–106+UU and U6 101–106+AU, assays were performed in Usb1 buffer containing 0–50 μM substrate using 2-fold dilutions (0.8, 1.6, 3.1, 6.2, 12.5, 25 and 50 μM) and 0.5 μM Usb1 at 37°C for 10 min. Time course experiments were used to confirm that all initial velocities were measured within the linear range. The products were analyzed by 20% polyacrylamide gel containing 8 M urea. Kinetic parameters and standard errors were determined from nonlinear regression fitting to the Michaelis–Menten equation using GraphPad software.

To investigate the effect of U6 secondary structure on Usb1 activity, 5′-end truncated and full-length U6 snRNAs, both containing with four additional uridine nucleotides at the 3′ end (U6 89–107+4U and U6 1–107+4U, respectively) were used. The reaction was carried out at 37°C for the indicated times in Usb1 buffer containing 2 μM Usb1 and 5 μM substrate RNA. 5′-FAM labeled U6 89–107+4U was used as a truncated RNA substrate. U6 1–107+4U was applied as a mixture containing 25 nM 5′-Cy5 labeled U6 1–107+4U and 5 μM non-labeled U6 1–107+4U. The products from U6 89–107+4U and U6 1–107+4U were analyzed by 20% acrylamide gel and 10% acrylamide gel containing 8 M urea, respectively.

### Computational methods

All molecular dynamics simulations used the program CHARMM (44) and classical interactions used the Charmm 36 all atom forcefield (45,46). Classical MD simulations took advantage of the CHARMM interface with OpenMM (47). Initial structures for simulations of human Usb1 were based on the crystal structures solved here. The Usb1–UU starting model was created by aligning the WT structure bound to UMP (PDB ID: 5V1M) to the Usb1-H208Q structure bound to 5′-UU-3′ and removing the UMP and Usb1-H208Q protein coordinates. The initial model for the Usb1–‘UA(*syn*)’ simulation was created by aligning the UA-bound H208Q structure with the wt–AMP structure, in which the adenosine is in the *syn* conformation and removing the coordinates for the 3′ U and Usb1-H208Q protein. The starting model for the ‘UA(*anti*)’ simulation was generated from *in silico* integration of an adenosine onto an equilibrated structure derived from the Usb1–UU structure described above. H120 was modeled to be singly protonated and H208 was modeled as doubly protonated.

The systems were initially equilibrated using classical MD, after which our general procedures for QM/MM simulations followed those we have used in our other recent studies of enzymes catalyzing phosphate cleavage (48,49,50,51). The systems were treated using the Generalized Solvent Boundary Potential (52) with a 25 Å inner region. QM/MM link atoms were added to the sidechains of residues in the QM region (Supplementary Figure S2). The QM region was treated at the DFTB3/3OB level (53,54,55,56) and the MM region was treated with the same force field as the classical MD simulations. A FIRES potential (57) prevented exchange of QM and MM water molecules during the MD simulations. The total system had ca. 7000 atoms, nearly all of which were in the inner region; the QM region contained 137 atoms and a total charge of –1. 1-, 2- and 3D free energy surfaces (FESs) were calculated through multi-walker metadynamics simulations using the program PLUMED (58) interfaced with CHARMM. Metadynamics parameters differed slightly for different sampling goals, but in general, Gaussians of height 0.1 kcal/mol and width 0.1 Å were deposited once every 200 timesteps (100 fs). For the ‘classic’ mechanism, three order parameters were subject to biasing potentials; these were the three antisymmetric stretches corresponding to: (i) O2′-H2′-NE2_H120_ (‘Nucleophile Deprotonation’), (ii) O2′-P-O5′ (‘Phosphoryl Transfer’) and (iii) NE2_H208_-HE2_H208_-O5′ (‘Leaving Group Protonation’). For the ‘triester-like’ mechanism, the nucleophile deprotonation coordinate was replaced with the ζ coordinate (59) to bias the center of excess charge, where 2′-O was the donor, a non-bridging oxygen was the acceptor and NE2_H120_ served as a proton shuttle. The other two coordinates were unchanged. Various trajectories (‘walkers’) to sample these coordinates were spawned either from equilibrium simulations or from other metadynamics simulations. For example, walkers for the three-dimensional (3D) metadynamics were taken from preliminary 1D and 2D simulations. The 3D simulations typically used 300–500 walkers, each of which was sampled for 0.5–1 ns. The final 3D PMFs, therefore, are the result of ca. 0.1–0.2 μs of QM/MM sampling (0.2–0.4 × 10^9^ timesteps) and constructed from ca. 10^6^ Gaussians. Solvent isotope effects were calculated using a path-integral free energy perturbation method (60). Additional details of all methods are available in the Supplementary Data.

## RESULTS

### Kinetics and substrate specificity of human Usb1

**Figure 2.**
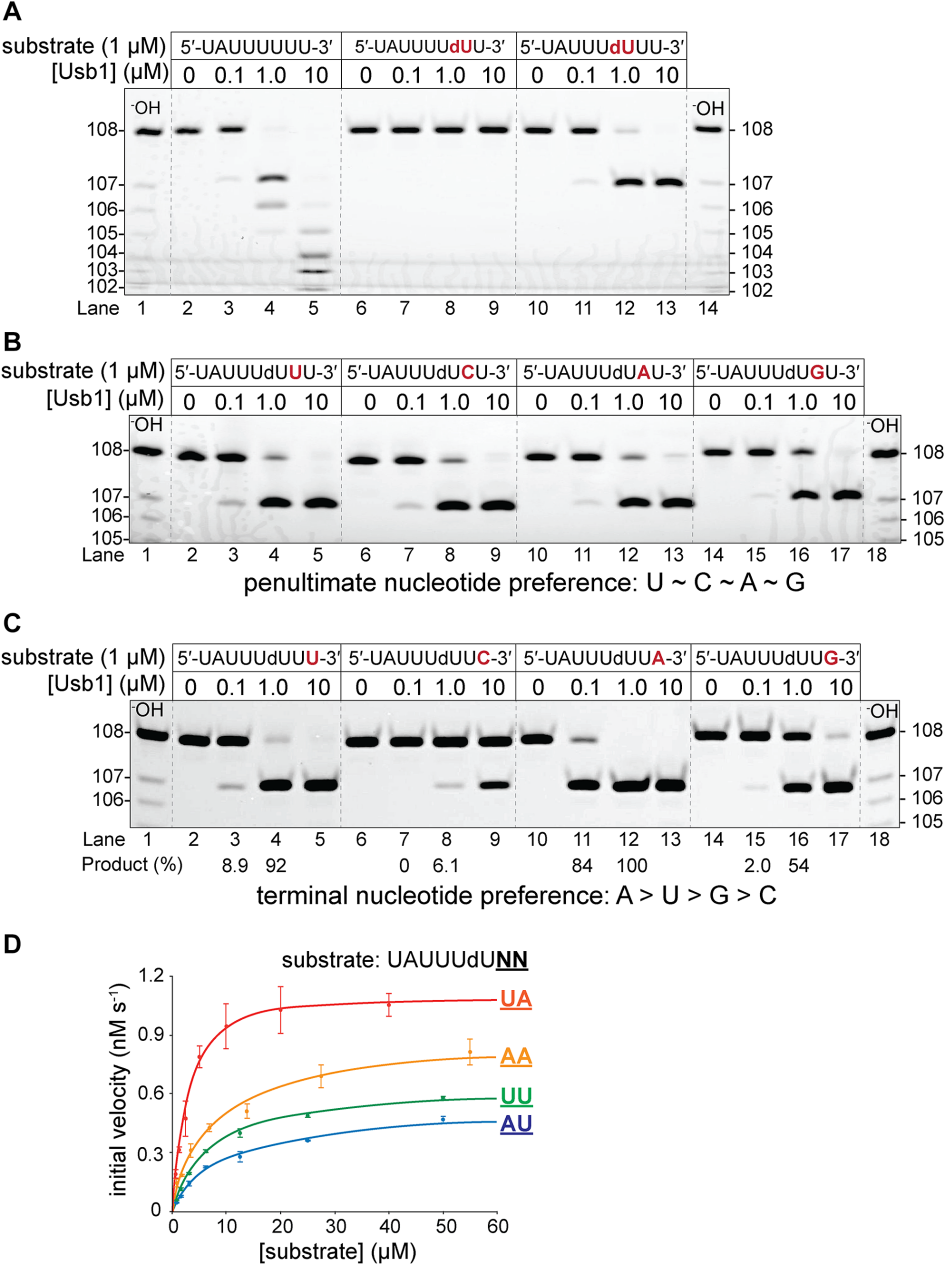
*In vitro* analyses of Usb1 activity. (**A**) A minimal substrate analog was used to monitor Usb1 3′ exoribonuclease activity (lanes 2–5). Substitution of the penultimate (*n–1*) uridine with 2′-deoxyuridine (dU) prevents Usb1 processing (lanes 6–9). Substitution of the antepenultimate (*n–2*) uridine with 2′-deoxyuridine results in formation of a single product (lanes 10–13). (**B**) Analysis of the *n–1* nucleotide preference for Usb1 processing, showing that the enzyme is mostly insensitive to the identity of the *n–1* nucleotide. (**C**) Analysis of the *n* nucleotide preference. The amount of product (%) in the presence of 0.1 and 1.0 μM Usb1 is indicated below the lane numbers. (**D**) Plots of steady-state kinetic analysis with adenosine or uridine at the *n–1* and *n* positions. Rates were measured for seven different substrate concentrations (see ‘Materials and Methods’ section) and error bars represent standard deviations obtained from two biological replicates for each of the seven substrate concentrations. Rate as a function of substrate concentration shows Michaelis–Menten kinetics for all substrates and fits to the Michaelis–Menten equation yielded *k*_cat_ and *K*_m_ (cf. Table 1).

Previous *in vitro* activity assays have shown that human Usb1 is a 3′-5′ exoribonuclease that can process different RNA sequences to various degrees (7,13,15). However, rigorous analyses of sequence specificity have been complicated by slow reaction kinetics and the distributive nature of Usb1 processing that result in the formation of multiple products (7,13,15). Indeed, using a fluorescently labeled oligonucleotide substrate corresponding to the 3′ fragment of U6 snRNA, we observe very little product accumulation after 1 h at 37°C with 1 μM substrate in the presence of 0.1 μM enzyme, and higher concentrations of enzyme result in heterogenous products (Figure 2A). Therefore, we developed a single cleavage RNA substrate in order to measure the kinetics and specificity of Usb1. Human Usb1 cleavage results in a 2′,3′-cyclic phosphate (6,7,10,13), and utilizes the 2′ oxygen directly preceding the scissile phosphate as a nucleophile. Therefore, when a 2′ deoxy modification is incorporated at the penultimate (hereafter, ‘*n–1*’) nucleotide, cleavage of the last nucleotide is blocked (Figure 2A). Moving the deoxynucleotide to the ‘*n–2*’ position results in single-cleavage substrates that yield single products (Figure 2A).

Using these single-cleavage substrates, we tested the importance of the *n–1* and terminal (hereafter, ‘*n*’) nucleotides for Usb1 processing. We found that the enzyme is mostly insensitive to the identity of nucleotides at the *n–1* position (Figure 2B). However, we observe a clear preference at the *n* position for adenosine over uridine, followed by guanosine and lastly, cytidine (Figure 2C). Considering the preferential processing of terminal adenosines, we hypothesized that Usb1 would display high activity for oligoA RNA substrates. Indeed, in the presence of excess (10 μM) enzyme, an oligoA substrate is completely degraded whereas oligoU is not (Supplementary Figure S3).

To gain further insight into the enzymatic mechanism, multiple turnover kinetic parameters were determined for substrates ending in UU, UA and AA by monitoring initial rates of product formation as a function of substrate concentration (Figure 2D and Table 1). These data reveal that Usb1 is most efficient at processing substrates terminating in UA. The *k*_cat_/*K*_m_ value for UA is twenty-fold higher than UU (Table 1).

**Table 1.** Kinetic parameters of Usb1 for single-cleavage substrates

Substrate	*k*_cat_ (10^–3^ s^–1^)	*K*_m_ (μM)	*k*_cat_/*K*_m_ (10^3^ M^–1^ s^–1^)	Relative^a^
UAUUUdUUU	1.31 ± 0.02	7.58 ± 0.38	0.17	1.0
UAUUUdUAU	1.07 ± 0.05	9.75 ± 1.22	0.11	0.63
UAUUUdUUA	11.85 ± 0.61	3.12 ± 0.56	3.80	22
UAUUUdUAA	8.78 ± 0.48	7.17 ± 1.21	1.23	7.2

dU is 2′-deoxyuridine.

Standard errors were obtained from nonlinear regression fitting to the Michaelis–Menten equation.

^a^Relative to the *k*_cat_/*K*_m_ value for UAUUUdUUU.

### Structures of human Usb1 with substrate RNA

**Figure 3.**
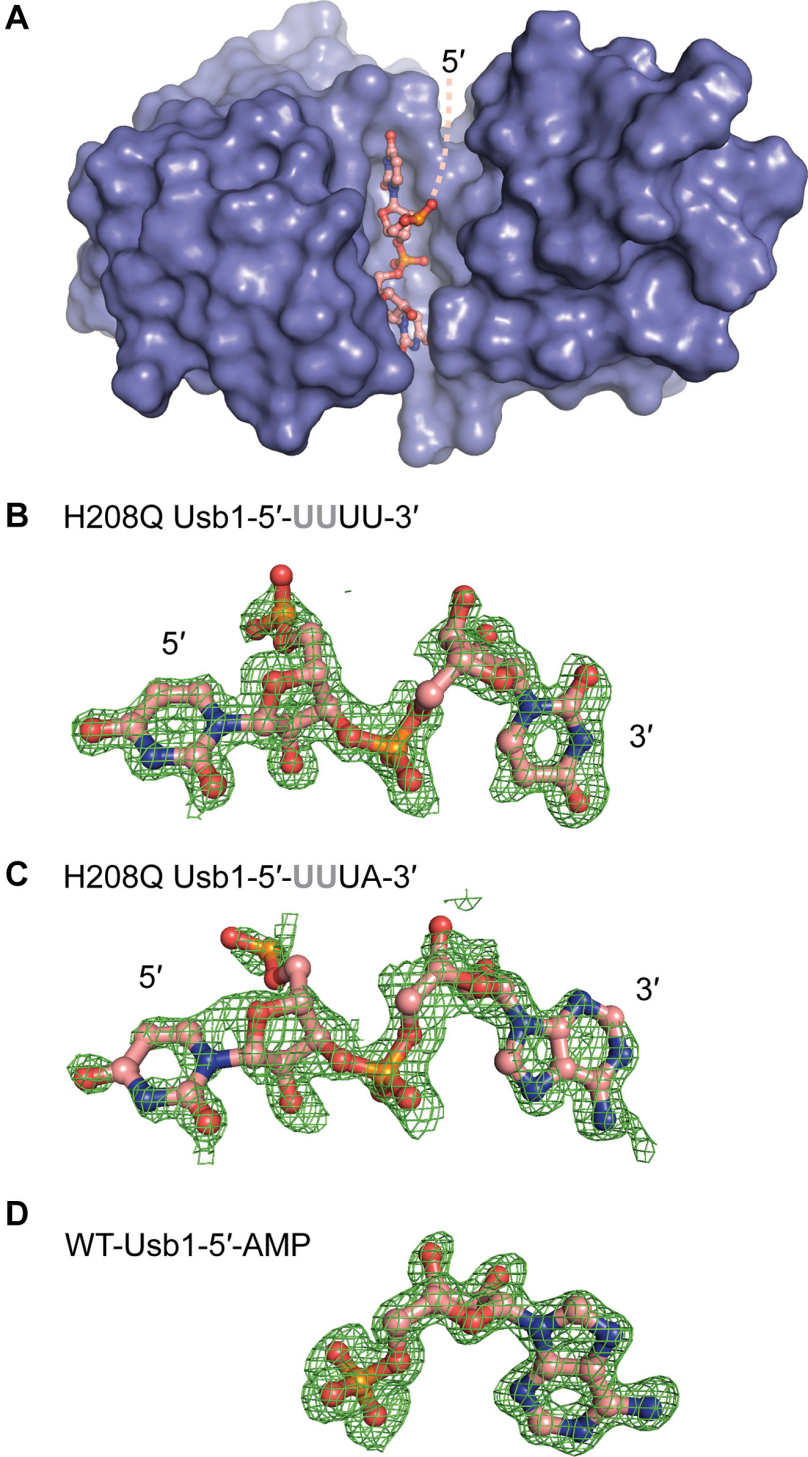
Structures of trapped enzyme–substrate complexes. (**A**) Architecture of a trapped enzyme–RNA complex using truncated Usb1 (residues 79–265) and tetrauridine. (**B**–**D**) Annealed omit maps for bound RNAs: (B) 5′-UUUU-3′; (C) 5′-UUUA-3′; (D) substrate analog, adenosine 5′-monophosphate. The m*F*_o_–D*F*_c_ electron density maps are countered at 2.5σ. The first two uridines (sequences depicted in gray) were disordered and not visible in the calculated electron density maps.

We determined co-crystal structures of Usb1 bound to RNA in order to clarify the enzyme′s catalytic mechanism and preferential activity toward 3′ adenylated U6 RNAs. We generated an inactive mutant of Usb1 (13,15) that could be co-crystallized with unmodified RNAs. Mutation of His208 was found to confer the strongest defect in catalysis (Supplementary Figure S4) and was therefore used for crystallization trials, leading to successful structure determination of mutant Usb1 (H208Q) bound to RNA oligonucleotides 5′-UUUU-3′ and 5′-UUUA-3′. Data collection and refinement statistics are shown in Table 2. The structures are isomorphous to the previously reported structures of human Usb1 in crystallographic space group *P*2_1_. Globally, Usb1 changes little upon substrate binding, as the average pairwise r.m.s.d. between the structures reported here and the previous structure of the free enzyme is 0.7 Å (13,15). The RNAs are bound in the central cleft of Usb1 (Figure 3A). Only the last two nucleotides of RNA are clearly visible in the electron density maps, and the first two nucleotides are presumed to be disordered. The two bound nucleotides adopt a conformation in which the nucleobases are splayed far apart from each other (Figure 3B and C).

**Table 2.** Data collection and refinement statistics

	H208Q-Usb1–UU PDB 6D30	H208Q-Usb1–UA PDB 6D2Z	WT-Usb1–5′ AMP PDB 6D31
**Data Collection**			
Wavelength (Å)	0.9787	0.9792	0.9792
Resolution range (Å)^a^	53.04–1.17(1.19–1.17)	44.53–1.18 (1.20–1.18)	44.39–1.20 (1.22–1.20)
Space group	*P*2_1_	*P*2_1_	*P*2_1_
Unit cell dimensions (Å)	41.70, 53.04, 45.81	41.39, 51.04, 46.30	41.43, 52.08, 46.24
	*β* = 105.72°	*β* = 105.88°	*β* = 106.26°
Total reflections^a^	233 514 (4655)	371 233 (8782)	366 062 (13 625)
Unique reflections^a^	60 998 (1882)	55 920 (2198)	57 826 (2757)
Multiplicity^a^	3.8 (2.5)	6.6 (4.0)	6.3 (4.9)
Completeness (%)^a^	94.1 (58.6)	91.6 (73.2)^b^	97.9 (94.1)
Mean I/σ(I)^a^	13.7 (1.0)	14.0 (1.0)	12.5 (1.3)
Wilson B-factor	12.33	12.47	14.02
R-merge^a^	0.044 (0.952)	0.052 (1.093)	0.056 (1.180)
CC_1/2_^a^	0.999 (0.331)	0.999 (0.547)	0.999 (0.679)
**Refinement**			
*R*_work_/*R*_free_^a^	0.15/0.17 (0.33/0.33)	0.15/0.17 (0.29/0.34)	0.15/0.18 (0.29/0.34)
Total number of atoms	3482	3219	3481
macromolecules	3200	2993	3218
ligands	62	65	50
water	220	161	213
RMS (bonds)	0.014	0.023	0.022
RMS (angles)	1.297	1.735	1.676
Ramachandran favored	97.31%	98.39%	98.39%
Ramachandran outliers	0%	0%	0%
Average B factor (Å^2^)	20.38	22.78	23.09
protein	19.58	21.93	22.36
ligands/ions	31.90	41.27	24.86
solvent	28.70	31.19	33.74

^a^Values shown in parentheses are for the highest resolution shell.

^b^Values of completeness are 94.0 (91.6) at resolution range between 44.53 and 1.30.

**Figure 4.**
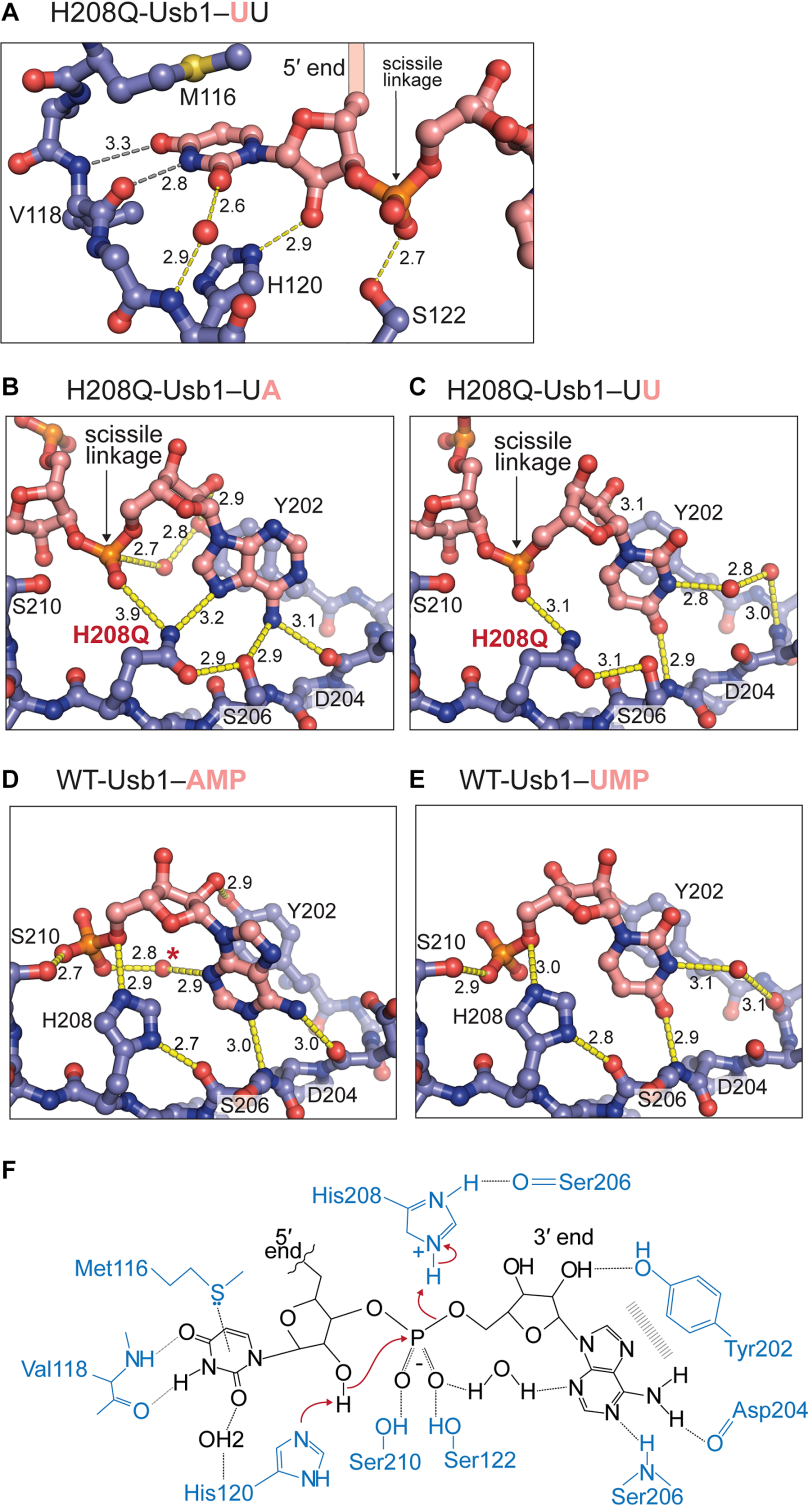
Contacts between Usb1 and substrate RNA. Hydrogen-bonding contacts are shown by yellow lines and are measured in Ångströms. (**A**) Structure of the *n–1* pocket for H208Q-Usb1 bound to UUUU. The dotted gray lines indicate possible weak hydrogen bonds due to their length and/or angle. (**B**–**E**) Structures of nucleotides bound in the *n* pocket. (B) H208Q-Usb1 bound to UUUA. (C) H208Q-Usb1 bound to UUUU. (D and E) Comparison of the terminal nucleotide binding mode in the *n* pocket of wild-type Usb1 structures. (D) WT-Usb1 bound to adenosine 5′-monophosphate. A buried water molecule is indicated with red asterisk. (E) Previously determined structure of WT-Usb1 bound to UMP, PDB ID: 5V1M. (**F**) Proposed mechanism of Usb1 catalyzed cleavage of a UA dinucleotide.

The *n–1* nucleotide binding pocket in both the H208Q-Usb1–UUUU and H208Q-Usb1–UUUA structures are nearly identical (r.m.s.d. = 0.3 Å). Both have the *n–1* uridine bound in the *n–1* nucleotide binding pocket with few contacts between the uridine nucleotide and Usb1, including an apparent n→π* contact (61) between Met116 and the uridine, a relatively long (3.3 Å) hydrogen bond between the O4 oxygen of uridine and the backbone nitrogen of Val118, a hydrogen bond with suboptimal angle between uridine N3 and the carbonyl oxygen of Val118, and a solvent-bridged hydrogen bond between the uridine O2 oxygen and the backbone nitrogen of His120 (Figure 4A). The structures show that His120 is positioned to function as a catalytic base by deprotonating the 2′ oxygen and facilitating an in-line attack on the scissile phosphodiester linkage.

The *n* nucleotide binding pocket reveals the H208Q mutation that was used to generate the trapped Usb1–RNA complex forms hydrogen bonds with a non-bridging oxygen of the labile phosphodiester and the sidechain of Ser206 in both the UA and UU bound structures (Figure 4B and C). Comparison of the *n* pocket binding mode in the H208Q mutant with the previously determined structure of wild-type Usb1 bound to uridine 5′-monophosphate (15) revealed a displacement of the phosphate group by 1 Å (Figure 4C and E; Supplementary Figure S5A). We therefore considered the possibility that the H208Q mutation perturbs the conformation of the scissile phosphate group and terminal nucleotide. In order to visualize how adenosine binds in the unperturbed active site, we crystallized wild-type Usb1 with the substrate mimic adenosine 5′-monophosphate (AMP, Table 2). AMP bound in the *n* pocket, with His208 well-positioned to act as a catalytic acid to protonate the leaving group 5′ oxygen of the terminal nucleotide (Figures 3D and 4D). His208 is also hydrogen bonded to the backbone oxygen of Ser206. Comparison of the wt-Usb1–AMP structure with the H208Q-Usb1–UUUA structure confirmed our hypothesis that the H208Q mutation perturbs the position of the scissile phosphate group and terminal nucleotide by ∼1 Å (Figure 4B and D; Supplementary Figure S5B). In the wt–AMP structure, Ser210 (part of the HxS catalytic motif) helps orient the scissile phosphate by forming a 2.7 Å hydrogen bond to one of the non-bridging oxygens. In the H208Q structures, Ser210 is 3.7 Å away and cannot hydrogen bond to the phosphate group (Figure 4B and D).

In all three structures reported here, the nucleobases in the *n* pocket stack on Tyr202, which also makes a hydrogen bond to the 2′ hydroxyl group of the terminal nucleotide in the *n* pocket (Figure 4B–D and F). Mutation of Tyr202 to alanine leads to a pronounced decrease in catalytic efficiency without altering the nucleotide specificity for Usb1, in accordance with its important role in stacking and hydrogen bonding (Supplementary Figure S6).

Interestingly, the adenosine in the AMP bound structure adopts a *syn* conformation that was not observed in any of the above structures. This conformation allows formation of hydrogen bonds between the N1 nitrogen of AMP and the backbone nitrogen of Ser206, and the AMP N6 nitrogen with the backbone oxygen of Asp204 (Figure 4D). The *syn* conformation also allows a water molecule to hydrogen bond to both the AMP N3 nitrogen and the non-bridging oxygen of the scissile phosphate (Figure 4D). Coordination of this water molecule may help stabilize an in-line conformation of the scissile phosphate. Comparison of the AMP-bound structure with our previously determined UMP-bound structure shows that UMP cannot coordinate a water molecule in a similar fashion. We also observe that, relative to the UMP bound structure, AMP makes a shorter hydrogen bond between the hydroxyl group of Ser210 and the scissile phosphate (Figure 4D and E). These unique features of AMP bound to the *n* pocket may provide a rationale as to why the enzyme is more efficient at cleaving a terminal adenosine relative to uridine.

### QM/MM simulation of the Usb1 enzymatic mechanism

**Figure 5.**
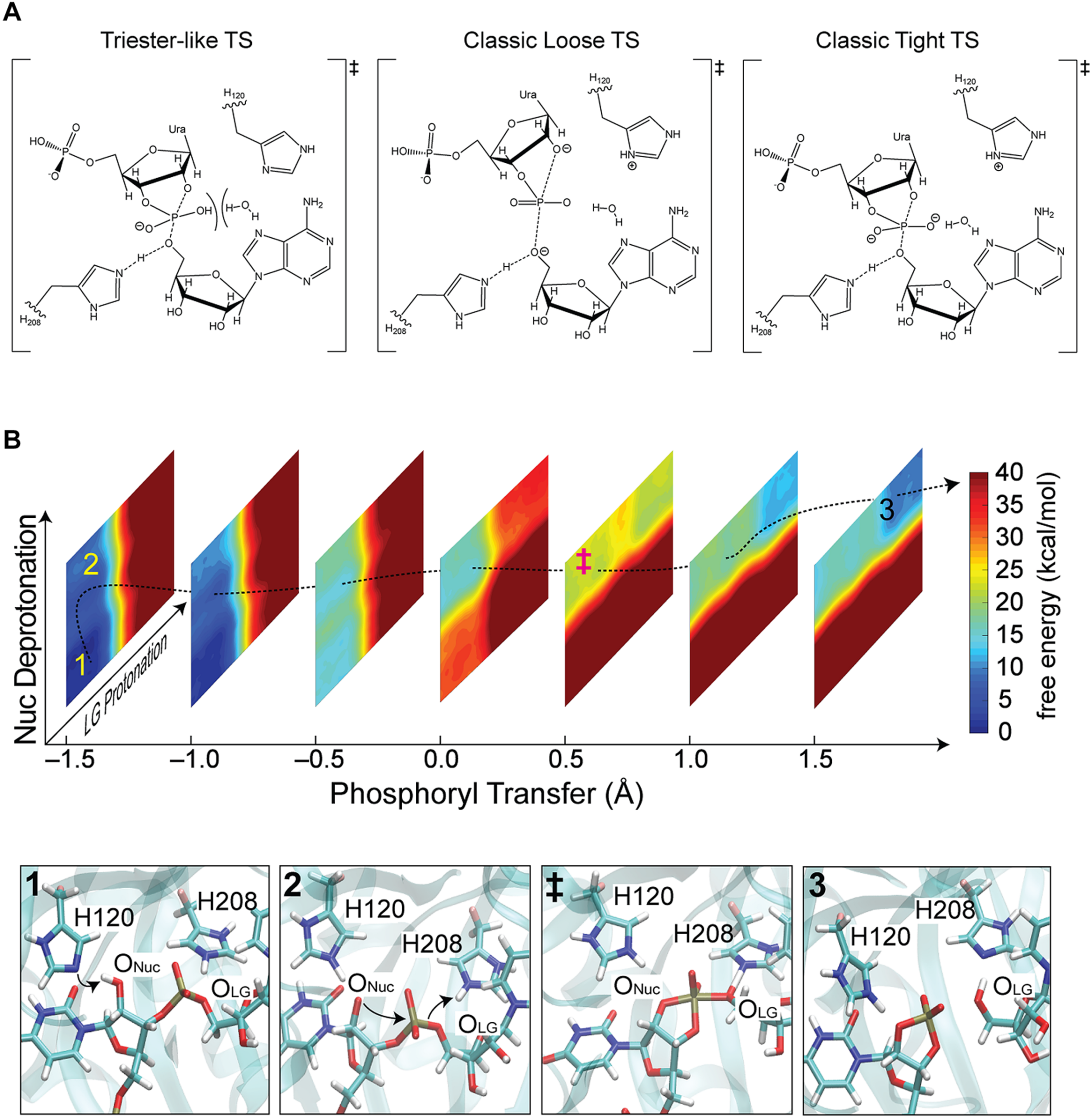
QM/MM calculations for the enzymatic reaction mechanism. (**A**) Schematic TS structures for different mechanisms of phosphoryl transfer. The structures differ substantially in the negative charge on the non-bridging oxygens and the ability of water to preferentially stabilize that charge versus the relevant charge in the ground state. In the triester-like TS, the additional proton on the non-bridging oxygen hinders any advantage offered by the water. In the loose version of the classic TS, negative charge on the non-bridging oxygens is greater in the ground state than in the TS (50), so an additional H-bond donated by water will be anti-catalytic. Only in the classic mechanism that uses a tight TS does additional negative charge accumulate on the non-bridging oxygens, making an additional H-bond—either from water or an active site residue—likely to be catalytic. We note that other TSs are possible; we show these three merely to illustrate the scope of possibilities. (**B**) 3D FES of the classic mechanism, displayed as 2D slices at different points along the phosphoryl transfer coordinate (A 3D rendering of this surface is shown in Supplementary Figure S11). Representative structures at different points along the minimum free energy path (dotted black line) are shown below and their locations are as labeled on the surface. Free energy is in kcal/mol. The surface implies a mechanism where deprotonation of the nucleophile (1→2) precedes the other two processes, but structure (2) is not necessarily an intermediate, as the barrier to return to (1) is quite small. Following deprotonation, nucleophilic attack and protonation of the leaving group are more synchronized and the system passes through a rate-limiting TS (‡) that involves motion along the phosphoryl transfer coordinate and some along the leaving group protonation coordinate.

We employed hybrid QM/MM simulations to test two primary mechanistic hypotheses for the reaction catalyzed by human Usb1, which we refer to as the ‘classic’ mechanism and the ‘triester-like’ mechanism (62), respectively. Starting models were derived from the crystal structures determined here, and simulations were performed for Usb1 bound to the 5′-UU-3′ substrate, as well as to the 5′-UA-3′ substrate where the initial structures contained the adenosine in both the *syn* and *anti* conformations. The ‘classic’ mechanism (Supplementary Figure S7A) involves three primary processes: (i) deprotonation of the 2′-OH of the *n–1* nucleotide by His120, (ii) nucleophilic attack on the phosphoryl group and (iii) protonation of the 5′-O of the *n* nucleotide (the leaving group) by His208. In testing this mechanism, we made no assumptions about the order of these events; we thoroughly sampled all three of these reaction coordinates to determine the minimum free energy path to reach products. The resulting FES for the 5′-UU-3′ substrate is presented in Figure 5B. The minimum free energy path on this surface indicates a mechanism where a modestly uphill deprotonation of the 2′-OH precedes nucleophilic attack. The deprotonated state is not an intermediate in the strictest sense; the barrier to returning to reactants is likely below the zero point energy of the N-H stretch, so it would have no finite lifetime. Following this deprotonation, the 2′-O^(–)^ attacks the phosphorus (P) while the 5′-O of the cleaved residue separates from the P. Concerted with this transphosphorylation, although not perfectly synchronized, is the protonation of the leaving group oxygen by His208. A movie of the process is available as Supplementary Movie S1 for the UU substrate. The minimum free energy path follows a ‘tight’ (Associative, or S_N_2-like) pathway (Figure 5A), where the nucleophile attacks prior to departure of the leaving group; transition state (TS) geometries are available in Supplementary Table S1. We have shown that this type of pathway results in greater negative charge on the phosphoryl group at the TS than in the ground state (50), which would enable the adenosine-coordinated water—or some other H-bond donor—to serve a catalytic role.

A concern that arose in the analysis of this mechanism was that the free energy barrier for the process was 21.6 kcal/mol, which is significantly larger than what one might expect for this type of reaction. Using TS theory, that barrier would correspond to *k*_cat_ of 0.5 × 10^–3^ s^–1^, which is far slower than other enzymes that catalyze the same kind of chemistry (63). Our recent work on a similar reaction (51) suggested that if anything, the QM method we used, DFTB3, *underestimates* barrier heights for this type of reaction. The *k*_cat_ value predicted by the QM/MM simulations is in good agreement with the measured values (Table 1).

Given the concern about the high barrier from QM/MM calculations, we also simulated an alternative ‘triester’ mechanism that has been proposed for ribonuclease A (Figure 5A; Supplementary Figures S7B and 8) (62). We conducted these simulations in such a way as to allow simultaneous sampling of the FES for both the classic and triester mechanisms (see Supplementary Data). The free energy barriers for the two mechanisms showed that the classic mechanism has a smaller barrier, indicating that the classic mechanism is more likely. Measurement of the kinetic parameters for the reaction (which were unavailable prior to completion of the simulations) found that our calculated rate for the classic mechanism is, in fact, strikingly similar to the measured rate (Table 1). Nonetheless, to thoroughly test the veracity of our simulations, we used the simulation of the classic mechanism to predict experimental observables in addition to the rate constant—both quantitative and qualitative. Based on the location of the TS, we reasoned that His208 plays a larger role in stabilizing the TS than His120. That is, since the proton transfer from 2′-OH to His120 is essentially complete at the TS, while the proton transferring from His208 to the leaving group is in flight at the TS, mutations of His208 would be more detrimental to catalysis than mutations to His120. This is in fact consistent with our analysis of mutant Usb1 activities (Supplementary Figure S4). More quantitatively, we calculated the H/D isotope effect on the proton that is in flight at the TS and obtained a value of 1.4. Furthermore, since our calculated TS involves exactly one proton that is in flight, the model predicts a linear relationship in a proton inventory experiment, which was confirmed experimentally (Supplementary Figure S9). The magnitude of the H/D isotope effect on *k*_cat_ was larger than our predicted value (2.7 versus 1.4), but measured magnitude is well within the typical range for a single proton transferred in the rate-limiting TS (64).

**Figure 6.**
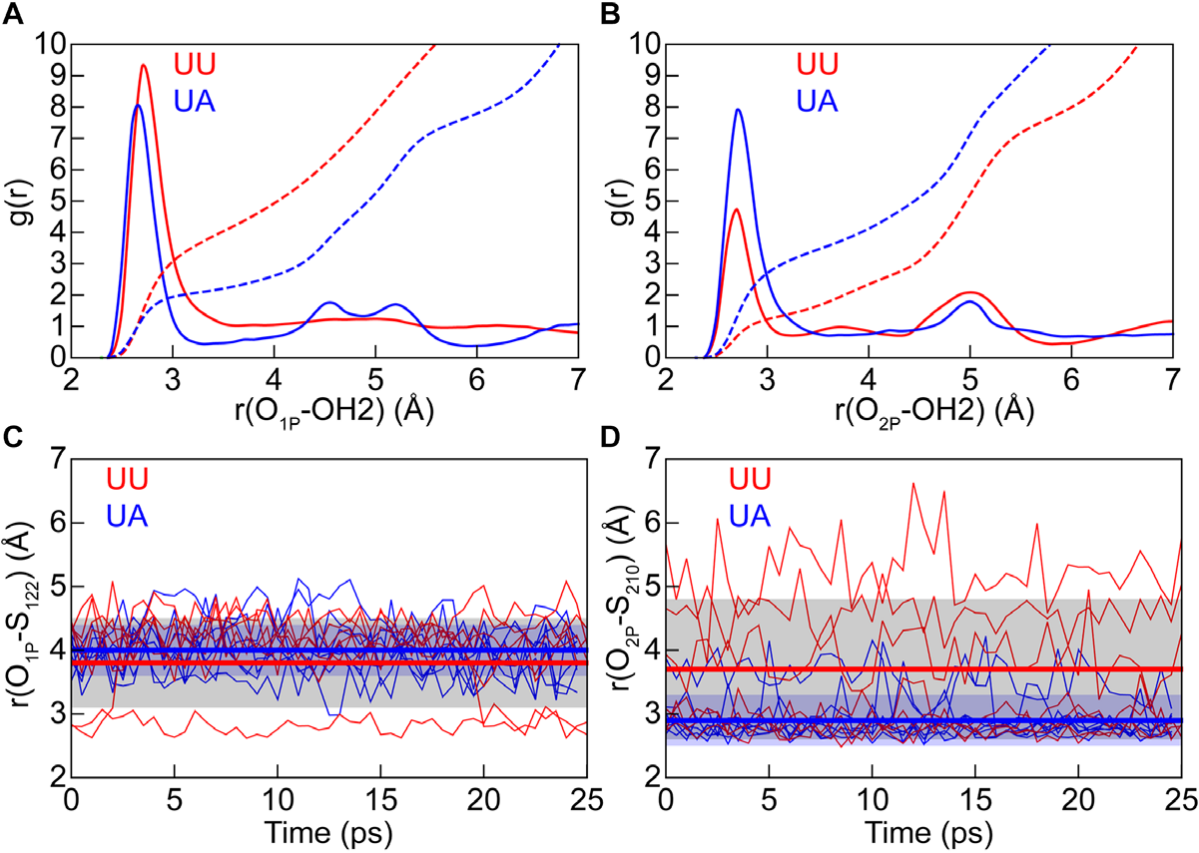
Interactions of non-bridging oxygens at the TS in simulations with UA(*syn*) versus UU. (**A** and **B**) Radial distribution functions (RDFs, solid lines) and integrated rdfs (dashed lines) surrounding the two non-bridging oxygens in simulations restrained to the TS region. The integration of the first solvation shell is larger for UU around O1P (A), but the effect is reversed around O2P (B), suggesting similar overall solvation of the two non-bridging oxygens. (**C** and **D**) Time courses of distances from the non-bridging oxygens to active site serines. Time courses are shown as thin lines for eight independent trajectories for each substrate. The thick lines indicate the average values during the simulations and the shaded regions indicate the root mean square fluctuations. While interactions between O1P and S122 are similar for the two substrates, those between O2P and S210 are significantly stronger for UA, confirming a role for increased H-bonding to the non-bridging oxygens during deadenylation.

The putative catalytic function of the water observed in the structure with AMP depends on the chemical and physical mechanism of the reaction (Figure 5A and Supplementary Figure S7) and thus mechanistic studies serve as a test of the proposed mode of discrimination between A and U. Specifically, for the observed active site water to function catalytically, the reaction must use a mechanism where the non-bridging oxygens accumulate more negative charge in the TS than in the ground state (Figure 5A); the wide space of mechanistic possibilities for biological phosphoryl transfer (65) make this requirement non-trivial. With the confirmation of the nature of the calculated mechanism and TS for 5′-UU-3′, we sought to test the role of water and H-bonding in preferential deadenylation. We conducted simulations with the substrate 5′-UA-3′ for both the syn ‘UA(*syn*)’ and anti ‘UA(*anti*)’ adenosine conformations observed here. A movie of the reaction of UA(*syn*) is available as Supplementary Movie S2. Following equilibration, the simulations were constrained to the TS of the classic mechanism and the level of hydration of the phosphate moiety was analyzed by calculating radial distribution functions (rdfs) for water surrounding atoms in the reactive phosphate (Figure 6). The UA simulations show differences versus UU in the hydration of the two non-bridging oxygens, but the differences are in the opposite directions for the two oxygens and balance one another. Interactions with active site serines, though, are better maintained in the UA(*syn*) simulation. Thus, while the simulations do not specifically support a role for additional water in UA, they do support a role for additional H-bonding by a combination of active site water and enzyme residues.

### U6 secondary structure inhibits Usb1 processing

**Figure 7.**
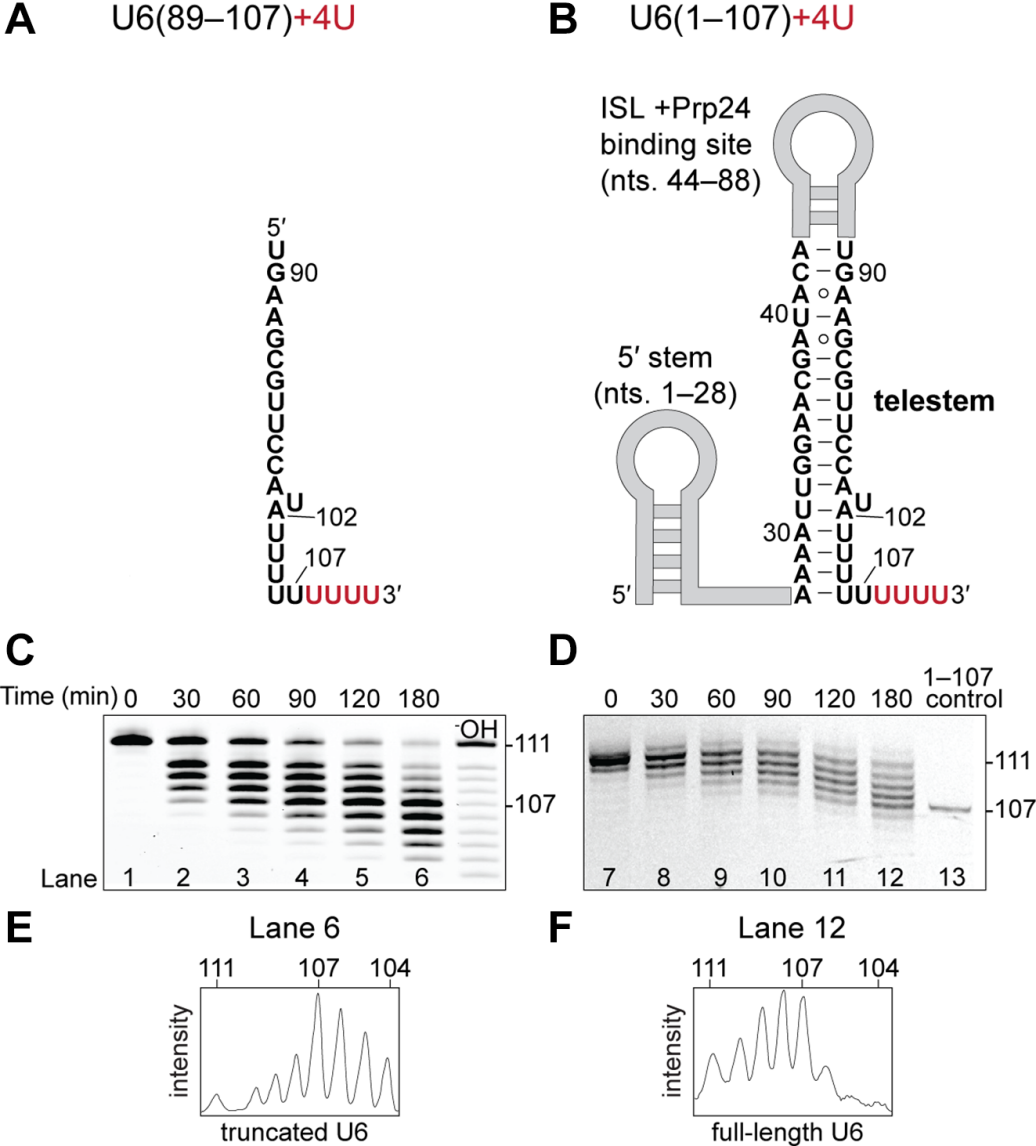
Effect of U6 secondary structure on Usb1 activity. (**A**) A single stranded fragment corresponding to the 3′ end of U6 snRNA, spanning nucleotides 89–107 with four additional uridines. (**B**) Schematic of full-length U6 snRNA with the base paired telestem sequence shown. (**C**) Usb1 activity assay using the single stranded fragment shown in (A). Usb1 is at 2 μM and RNA at 5 μM. (**D**) Usb1 activity assay with full-length U6 snRNA shown in (B). Conditions are identical to those in (C). (E) Quantification of lane 6 products from panel (C). (F) Quantification of lane 12 products from panel (D).

The Usb1 active site is only wide enough to accommodate two nucleotides of single stranded RNA (Figure 3A). The yeast and human versions of U6 snRNA are highly homologous (66), and our previous structures of yeast U6 snRNA bound by Prp24 (67,68) and in the U6 snRNP (69) showed formation of a helix near the 3′ end of U6 snRNA, termed the telestem. The telestem pairing region is relatively short in yeast, and potentially longer in human U6 snRNA (Figure 7B). We therefore asked if U6 snRNA secondary structure can regulate the extent of Usb1 processing. To test this hypothesis, we compared the extent of Usb1 processing of full-length (107 nucleotides + 4 extra U’s) U6 snRNA to a shorter single stranded sequence corresponding to the 3′ end of U6 with 4 extra U’s. We observe that the enzyme processes further into the single stranded substrate than full-length U6 snRNA (Figure 7C–F). Moreover, with full-length U6 snRNA, the enzyme exhibits minimal activity past the telestem region after 3 h of incubation. Therefore, we suggest that, at least in the absence of other protein cofactors, the major form of U6 snRNA corresponds to 107 nucleotides because positions 107 and 108 are the last available unpaired nucleotides for binding into the Usb1 active site.

## DISCUSSION

Our observation that human Usb1 preferentially cleaves the dinucleotide UA is consistent with the hypothesis that the enzyme removes terminal adenosines from U6 snRNA *in vivo*, and provides a molecular explanation as to why U6 snRNAs from cells lacking Usb1 are adenylated (13,20). To our knowledge, the affinity of the Lsm2–8 complex for monoadenylated U6 snRNA has not been reported. If 3′ adenylated U6 snRNA were to be incorporated into spliceosomal complexes, Usb1 might remove the terminal adenosine either during or after splicing catalysis. Recent cryo-EM studies of human spliceosomal complexes have achieved remarkable resolutions down to 3.4 Å (70); but even in these datasets, the density for the 3′ end of U6 snRNA is very fragmented and cannot be accurately modeled at the nucleotide level. Owing to the catalytic parameters reported here, recruitment of Usb1 to the spliceosome may be required for efficient processing of U6 snRNA. There is evidence that U6 snRNA is shortened within the spliceosome concomitant with splicing (71). Recruitment of Usb1 to the spliceosome may occur through its conserved N-terminal domain, which does not contribute to catalytic function but is essential for growth in *Saccharomyces cerevisiae* (15). This N-terminal domain is serine and proline-rich and may mediate protein–protein or protein–RNA contacts to help recruit Usb1 to the spliceosome. Consistent with this, high-throughput interactome studies (72,73,74,75) have identified potential binding partners of yeast Usb1 that include splicing factors such as Prp19 and Syf1 which reside near the 3′ end of U6 snRNA in post-catalytic spliceosomes (76,77). Recruitment of Usb1 to the spliceosome may ‘mark’ specific copies of U6 that are competent for splicing progression, thereafter protecting U6 from binding inhibitory proteins or encountering other exoribonucleases during spliceosome recycling.

While all RNA pol III transcripts terminate in 3′ oligouridine tails that could be potential substrates for Usb1, Usb1 is specific for U6 and U6atac snRNAs *in vivo*, with the exception of vault RNA, which also appears to be a substrate (20). This specificity may be related to the fact that newly synthesized 3′ ends of RNA pol III transcripts are tightly bound by the La protein, which likely prevents Usb1 processing. Therefore, Usb1 access to RNA 3′ ends may be regulated *in vivo* by RNA–protein interactions and/or RNA folding. For example, we have shown that the *S. cerevisiae* La homology protein (Lhp1) binds tightly to the unmodified U6 3′ end with low nanomolar affinity, but is displaced from U6 by the binding of Prp24 to the center of the RNA (15). Once U6 is modified by Usb1, it can no longer bind Lhp1 due to the terminal phosphate group, which aids in recruitment of the Lsm2–8 proteins (15). Thus, it is likely that a major function of human Usb1 is to prevent La from binding the U6 snRNA 3′ end, while promoting the binding of Lsm2–8.

Our proposed catalytic mechanism for Usb1 is similar to the previously proposed model based on the free protein structure (13), with some important differences. The previous study incorrectly proposed a role for Tyr161 stacking on the penultimate nucleotide and a cationic role for Arg168 interacting with the cyclic phosphate product (13). We observe that upon RNA binding, Tyr202 stacks on the penultimate nucleotide (Figure 4) and Arg168 is rotated 13 Å away from the catalytic cleft (Supplementary Figure S10C). As shown in this study, Usb1 is a relatively inefficient enzyme *in vitro*. However, with a *k*_cat_ of 10^–3^ s^–1^, Usb1 is still 10^6^-fold faster than the uncatalyzed rate of UpA hydrolysis (78). RNase A catalyzes an identical cleavage reaction via two catalytic histidine residues (79), and has a *k*_cat_ that is 10^6^-fold faster than Usb1 (78,80). We hypothesize that Usb1’s relatively slow *k*_cat_ may be due to the lack of a cationic group near the scissile phosphate. Previous studies have shown that positively charged residues Lys41 and His12 in RNase A stabilize the accumulation of negative charge on the phosphoryl group at the TS (Supplementary Figure S10A), and point mutations of Lys41 lead to a significant decrease in activity (81,82,83,84). *Escherichia coli* ThpR, an ortholog of Usb1, has a highly conserved arginine (Supplementary Figure S10B) required for activity (32). Thus, we hypothesize that Usb1 has a low *k*_cat_ due to the absence of a positively charged amino acid that can electrostatically stabilize the TS. The overall catalytic efficiency of Usb1 may be significantly improved by recruitment of the enzyme to the spliceosome, although we note that the previously observed U6 shortening activity on spliceosomes was observed after 180 min (71).

While Usb1 lacks a vicinal cation, it employs a catalytic mechanism that allows a unique property of adenosine to accelerate cleavage of that residue and therefore specifically prune terminal adenosines from U6 snRNA. The reaction proceeds through a ‘tight’ TS, where negative charge accumulates on the non-bridging phosphoryl oxygens. With no enzymatic cation available to stabilize the additional charge, an adenosine-coordinated water—or perhaps an enzymatic hydroxyl group—has a greater impact in specifically stabilizing the TS for cleavage of that residue. Thus, the nuances of the catalytic mechanism provide a basis for discrimination among different residues. This may be a more general phenomenon, as it has also been proposed, for example, in ‘transcript-assisted transcriptional proofreading’ by RNA polymerase (85).

The two nucleotides bound in the active site cleft of Usb1 have their bases splayed far apart from each other. This conformation facilitates the in-line attack geometry required for catalysis and similar conformations have also been observed in an RNase A–dinucleotide complex (86), and in ribozymes (87,88,89). For the H208Q Usb1 structures bound to UU and UA, both ribose sugar puckers on either side of the scissile phosphate are C2′ endo. The *syn* conformation of adenosine observed in the wt Usb1–AMP structure is also reminiscent of the RNase A–dinucleotide complex (86) and the hairpin ribozyme (87), both of which have *syn* nucleotides directly 3′ to a scissile phosphate.

Previous work on human Usb1 led to the hypothesis that the enzyme possesses 3′ end-measuring activity (13). It was proposed that the enzyme measures the presence of an adenosine 5 nucleotides upstream of the final cleavage site, which induces the enzyme to pause in order to produce the mature form of U6 snRNA found in cells (13). However, this hypothesis does not agree with the kinetic and structural data presented here. Under our assay conditions, recombinant Usb1 fails to demonstrate 3′ end-measuring activity with either short or long RNA substrates (Figures 2A and 7C; Supplementary Figures S3 and 4). We note that the previous activity assays contained either equal or excess concentrations of enzyme over substrate and were in low ionic strength buffer (20 mM Tris, pH 8.0 and 1 mM dithiothreitol (DTT)) (13), whereas our kinetic measurements included conditions of substrate in excess of enzyme and employed a higher ionic strength buffer (100 mM NaCl, 20 mM HEPES, pH 7.5, 1 mM EDTA and 1 mM TCEP-HCl), and are closer to the pH optimum for the enzyme (Supplementary Figure S1). We speculate that the previously observed end-measuring activity may have been due to kinetic interference from multiple copies of Usb1 associating with the RNA. Furthermore, our data indicate that an end-measuring activity is not required to prevent over-processing, as the presence of secondary structure in full-length U6 snRNA is sufficient to prevent over-processing and can account for the observed length of mature U6 snRNA (Figure 7B and D). In activated spliceosomes, the U2/U6 helix II secondary structure also forms at the 3′ end of U6 (90) and may similarly serve to prevent Usb1 from over-processing U6 as well.

## DATA AVAILABILITY

Coordinates and structure factors have been deposited in the Protein Data Bank with accession codes 6D2Z, 6D30 and 6D31. Other data supporting the findings of this manuscript are available from the corresponding author upon request.

## Supplementary Material

Supplementary Data
