# Supplementary material for: Structural and mechanistic basis for preferential deadenylation of U6 snRNA by Usb1
Source: Nucleic Acids Res. 2018 Sep 12;46(21):11488–501. doi: 10.1093/nar/gky812 (PMC6265477; doi:10.1093/nar/gky812)
Supplement: Supplementary Data [file gky812_supplemental_files.zip › NAR-Suppl-0826.docx]

Supplementary data

**Structural and mechanistic basis for preferential deadenylation of U6 snRNA by Usb1**

Yuichiro Nomura^1,a^, Daniel Roston^1,3,a^, Eric J Montemayor^1,2^, Qiang Cui^3,^*, and Samuel E Butcher^1,^*

^1^ Department of Biochemistry, University of Wisconsin, Madison, Wisconsin 53706, USA

^2^ Department of Biomolecular Chemistry, University of Wisconsin, Madison, Wisconsin 53706, USA

^3^ Department of Chemistry, University of Wisconsin, Madison, Wisconsin 53706, USA

^a^ Equal contribution

* To whom correspondence should be addressed. Tel: +1 608 263 3890; Email: [sebutcher@wisc.edu](mailto:sebutcher@wisc.edu)

Correspondence may also be addressed to Qiang Cui. Tel: +1 617 353 6189; Email: [qiangcui@bu.edu](mailto:qiangcui@bu.edu)

Present Address: Qiang Cui, Department of Chemistry, Boston University, Boston, MA 02215

**Simulation of a potential triester mechanism**

An alternative mechanism, referred to as the “triester" mechanism (Supplementary Figure S7B), has one major difference vs. the classic mechanism: once His120 deprotonates the 2ʹ-OH, the proton does not remain with His120. Instead, His120 acts as a shuttle to transfer the proton onto one of the non-bridging oxygen atoms. The intuitive rationale that would favor this type of mechanism is the fact that phosphate triesters are more susceptible to hydrolysis than diesters (1), because the negative charge accumulation at the TS is less costly for the neutral triester vs. the anionic diester. Thus, protonating the non-bridging oxygen reduces the negative charge on the phosphoryl group so that it more closely resembles a triester, possibly making it more reactive.

To test this mechanism, we employed a method developed to model long range proton transfer from a defined donor to a defined acceptor, which allows other groups to participate in shuttling the proton from donor to acceptor (2). For these simulations we defined the 2ʹ-O as the donor and the proximal non-bridging oxygen as the acceptor, while allowing His120 to shuttle the proton from donor to acceptor. This reaction coordinate, ζ, is defined based on the center of excess charge (i.e., the position of the proton) relative to the donor and acceptor, such that when the proton is bound to the donor ζ ≈ –1, and when bound to the acceptor, ζ ≈ +1. In conformations where the proton is bound neither to donor, nor acceptor—as when it is bound to His120—then ζ has a value between –1 and 1. The other two reaction coordinates, namely the phosphoryl transfer and the leaving group protonation, remained the same as in the initial simulations of the classic mechanism. Importantly, the nature of the ζ coordinate in these simulations allows the system to sample both the classic mechanism and the triester mechanism, without artificially biasing one or the other (Supplementary Figure S8). That is, the entire span of the coordinates describing phosphoryl transfer and leaving group protonation can be sampled while the proton is bound to the non-bridging oxygen and while it is bound to His120.

The triester mechanism predicts that the FES would contain a stationary point (either intermediate or TS) in the region near ζ = +1. We searched for such a point by numerically minimizing the gradient of the FES with initial guesses in the range ζ = 0.8–1.0 using the program *Mathematica* (3). We located a point at ζ = +0.96 with gradient converging to 0; the other coordinates at that point indicated progress along the phosphoryl transfer coordinate is near the halfway point and little progress has been made in protonating the leaving group (Supplementary Figure S8 right). We also located a point at ζ = –0.18 with gradient converging to 0 (Supplementary Figure S8 middle). Importantly, this stationary point was lower in free energy than the one at ζ = +0.95 by around 6 kcal/mol. By conducting simulations restrained to the stationary point at ζ = –0.18, we found that it was the same TS that we identified in the initial simulations; the proton was bound to His120 and reaction progress in the other two coordinates was nearly identical in the two simulations. Geometric properties of the TSs found by different means are listed in Supplementary Table S1.

**MATERIALS AND METHODS**

**Sequences of Protein-Coding ORF and Oligonucleotides**

Codon-optimized full-length *Homo sapiens* Usb1 (hUsb1) ORF for expression in *E. coli*:

ATGTCAGCTGCACCATTAGTCGGGTATTCATCATCGGGTTCTGAGGATGAGTCCGAGGACGGGATGCGTACACGTCCTGGCGACGGATCGCACCGTCGTGGGCAGTCCCCATTGCCTCGCCAGCGCTTCCCTGTGCCCGATTCGGTCCTGAACATGTTTCCAGGGACCGAGGAAGGACCGGAAGATGATAGCACTAAGCATGGAGGTCGCGTACGCACTTTCCCACACGAGCGCGGAAACTGGGCTACACATGTGTACGTTCCTTACGAAGCGAAGGAAGAATTCCTGGATTTGCTTGATGTCTTGCTTCCACATGCGCAGACTTACGTGCCGCGCTTAGTCCGCATGAAAGTCTTTCACCTGTCCTTGTCCCAATCGGTAGTTCTTCGCCATCATTGGATTTTGCCTTTTGTGCAGGCGTTGAAGGCCCGTATGACATCGTTCCACCGCTTTTTTTTCACCGCCAATCAGGTAAAGATTTATACAAATCAAGAGAAGACACGCACATTCATTGGCTTGGAGGTAACCTCAGGGCACGCTCAATTTTTGGACTTAGTAAGTGAAGTAGACCGCGTGATGGAGGAGTTCAACCTTACAACTTTCTATCAAGATCCGTCCTTCCACCTGAGCTTAGCCTGGTGCGTGGGAGATGCTCGCCTTCAGTTGGAAGGCCAATGCCTTCAAGAACTGCAAGCAATCGTAGACGGGTTCGAGGACGCCGAGGTACTGCTTCGTGTACACACCGAGCAGGTTCGCTGCAAATCAGGTAATAAGTTTTTCTCCATGCCACTTAAATGA-3′

Truncated Usb1 forward primer: 5′-GGAAACTGGGCTACACATGTGTACGTTCCT-3′

Truncated Usb1 reverse primer: 5′-CATATGAGCGCCCTGGAAATACAGGTTTTC-3′

H120N-Usb1 forward primer: 5′-AACCTGTCCTTGTCCCAATCGGTAGTTCTTCGCCATCATTGG-3′

H120N-Usb1 reverse primer: 5′-AAAGACTTTCATGCGGACTAAGCGCGGCACGTAAGTCTGC-3′

S122C-Usb1 forward primer: 5′-TGCTTGTCCCAATCGGTAGTTCTTCGCCATCATTGGATTTTGC-3′

S122C-Usb1 reverse primer: 5′-CAGGTGAAAGACTTTCATGCGGACTAAGCGCGGCACGTAAGTC-3′

Y202A-Usb1 forward primer: 5′-GCGCAAGATCCGTCCTTCCACCTGAGCTTAGCCTG-3′

Y202A-Usb1 reverse primer: 5′-GAAAGTTGTAAGGTTGAACTCCTCCATCACGCGGTC-3′

H208N-Usb1 forward primer: 5′-AACCTGAGCTTAGCCTGGTGCGTGGGAGATGCTCGCCTTCAG-3′

H208N-Usb1 reverse primer: 5′-GAAGGACGGATCTTGATAGAAAGTTGTAAGGTTGAACTCCTCC-3′

H208Q-Usb1 forward primer: 5′-CAGCTGAGCTTAGCCTGGTGCGTGGGAGATGCTCGCCTTCAG-3′

H208Q-Usb1 reverse primer: 5′-GAAGGACGGATCTTGATAGAAAGTTGTAAGGTTGAACTCCTCC-3′

S210C-Usb1 forward primer: 5′-TGCTTAGCCTGGTGCGTGGGAGATGCTCGCCTTCAGTTGG-3′

S210C-Usb1 reverse primer: 5′-CAGGTGGAAGGACGGATCTTGATAGAAAGTTGTAAGGTTG-3′

U6 1–106 forward primer: 5′-AGCATGGCCCCTGCGCAAGGATGACACGCAAATTCGTGAAGCGTTCCATATTTTGGGTCGGCATGGCATCTCC-3′

U6 1–106 reverse primer: 5′-AATCTTCTCTGTATCGTTCCAATTTTAGTATATGTGCTGCCGAAGCGAGCACCTATAGTGAGTCGTATTAGAA-3′

U6 1–107 forward primer: 5′-GGGTCGGCATGGCATCTCCACCTCCTCGCG-3′

U6 1–107 reverse primer: 5′-AAAAATATGGAACGCTTCACGAATTTGCGT-3′

U6 12–107 forward primer: 5′-GGCAGCACATATACTAAAAT-3′

U6 12–107 reverse primer: 5′-TATAGTGAGTCGTATTAGAA-3′

U6 12–107+4U forward primer: 5′-TTTTGGGTCGGCATGGCATCTCCACCTCCTCG-3′

U6 12–107+4U reverse primer: 5′-AAAAATATGGAACGCTTCACGAATTTGCGTGTC-3′

U6 splinted ligation oligo: 5′-TTTTAGTATATGTGCTGCCGAAGCGAGCAC-3′

**Kinetic Isotope Effects on the Cleavage Reaction by Usb1.**

In proton inventory experiments, U6 101–106+UA was used as a substrate. Assays were carried out at 37°C for 10 min in Usb1 buffer containing 0–50 µM substrate in the presence of 0.1 µM Usb1 with varying mole fractions of D_2_O:H_2_O (25:75, 50:50, and 75:25) and 0.2 µM Usb1 with 100% D_2_O, respectively. The conversion of pH from pD was calculated using equation: pD = 0.929 pH_obs_ +0.41 (4).

**Additional Computational Methods**

Initial structures for each system were solvated in a 78 Å cubic box of water with NaCl at a concentration of 150 mM using the CHARMM-GUI interface (5). These systems contained ca. 45,000 atoms. Each solvated system underwent a short geometry optimization and was subsequently heated from 48 K to 298 K during 125 ps with 1 fs timesteps using periodic boundary conditions. SHAKE was used to constrain all bonds to hydrogen atoms. Non-bonded interactions were cut off at a distance of 12 Å with a switching function from 10 Å to 12 Å. Electrostatic interactions were treated with the particle mesh Ewald method with 90 grid spaces in each direction. Each system was equilibrated at 298 K for at least 1 ns prior to any data collection. Production simulations were done in the NPT ensemble using the Andersen thermostat and monte carlo barostat available through the CHARMM/OpenMM interface.

Equilibrated structures from the classical MD simulations were used as starting points for hybrid QM/MM simulations. Our general procedures for QM/MM simulations followed those we have used in our other recent studies of enzymes catalyzing phosphate cleavage (6-9). The geometries of the equilibrated structures were optimized in the same conditions as the classical MD simulations, at which point water molecules were deleted outside of a 25 Å radius from the position of the reactive P atom. For all subsequent work, the system was treated using the Generalized Solvent Boundary Potential (GSBP). Atoms inside the 25 Å inner region were fully flexible during dynamics simulations; atoms between 25–27 Å were treated as a buffer region and those outside 27 Å were frozen.

After brief geometry optimizations, the systems were heated from 48 K to 298 K during 150 ps with 1 fs timesteps and equilibrated at that temperature for at least 100 ps prior to any data collection. SHAKE was used to constrain all bonds to hydrogen atoms during equilibration simulations. At least 10 ps prior to collecting any data during production simulations, the timestep was reduced to 0.5 fs and SHAKE constraints were removed from the two protons undergoing acid-base chemistry in the active site.

*Calculation of Isotope Effects***.** Solvent isotope effects were calculated using a path-integral free energy perturbation (PI-FEP) method (10). This method recovers the quantum behavior of nuclei by treating the nucleus as a string of quasi-particles, or “beads”; the configuration of the beads undergoes Monte-Carlo (MC) sampling within a given classical configuration. In the present work, we used similar sampling strategies as in our work on alkaline phosphatase (7,8). Once the converged 3D PMF was available, we conducted simulations harmonically restrained to the ground state (10) and the transition state using 5–10 walkers from the metadynamics simulations; we chose walkers based on their proximity to the ground state or TS at the end of the metadynamics simulations. Those simulations were equilibrated in the ground state or TS for 10 ps, followed by 50–100 ps production runs where frames were saved every 10 fs for PI-FEP analysis. For PI sampling the protons were treated by 32 beads and the donor and acceptor heavy atoms were treated by 16 beads each. The bead configurations were sampled with 10 MC steps in each classical configuration, for a total of ca. 500,000 quantum configurations in each state.


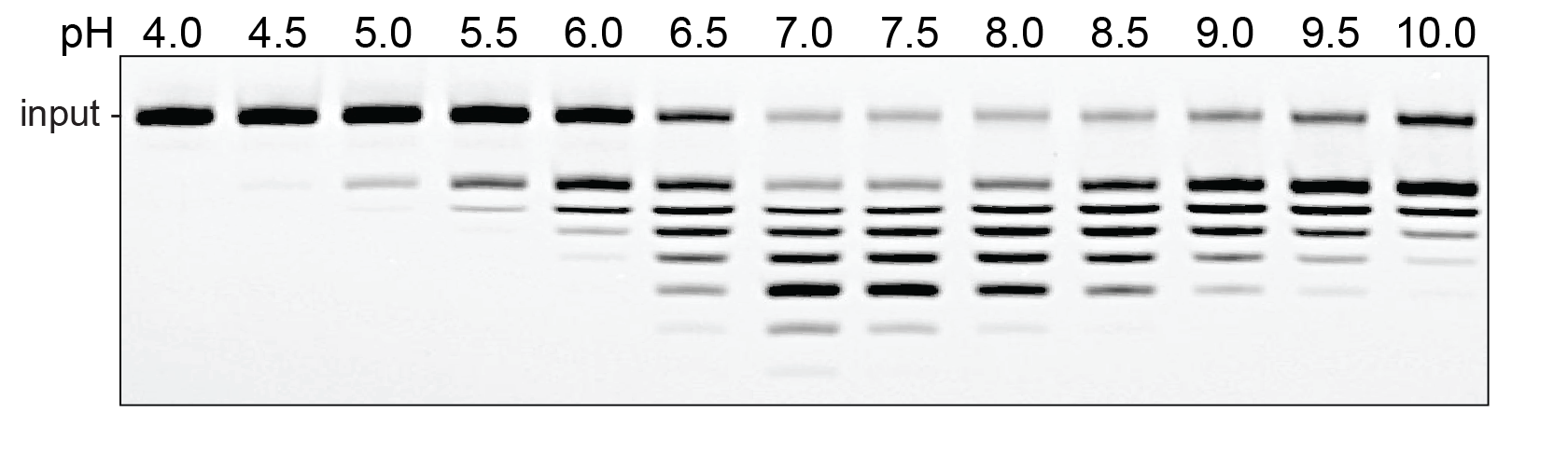


**Supplementary Figure S1.** Activity-pH profile for Usb1. Usb1 activity was measured at a wide range of pH values (4.0–10.0) using a buffer mixture containing 4 mM each of acetate, bis-tris, HEPES, Tris and CHES. Assays was carried out at 37°C for 5 min using 0.1 µM substrate in the presence of 0.5 µM enzyme.


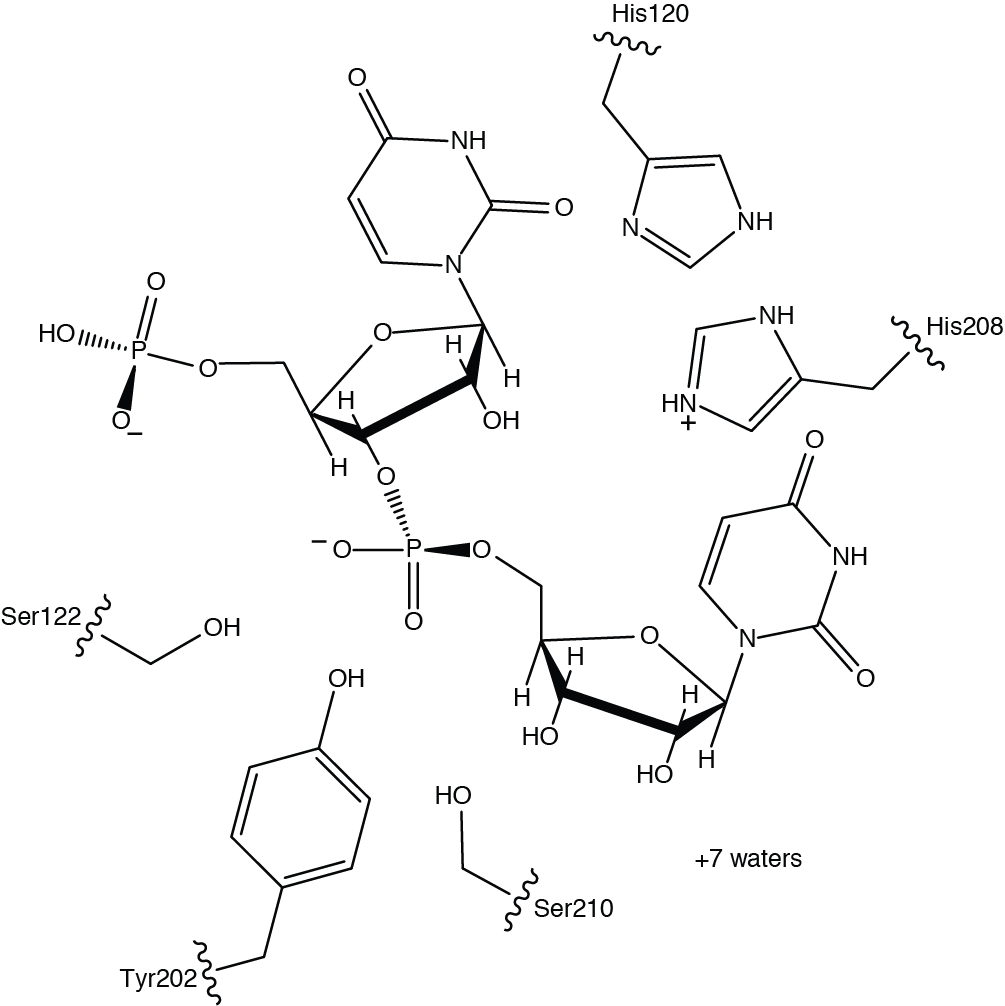


**Supplementary Figure S2.** The QM region for the 5ʹ-UU-3ʹ substrate. Simulations with 5ʹ-UA-3ʹ used the same QM region, including the entire Ade base.


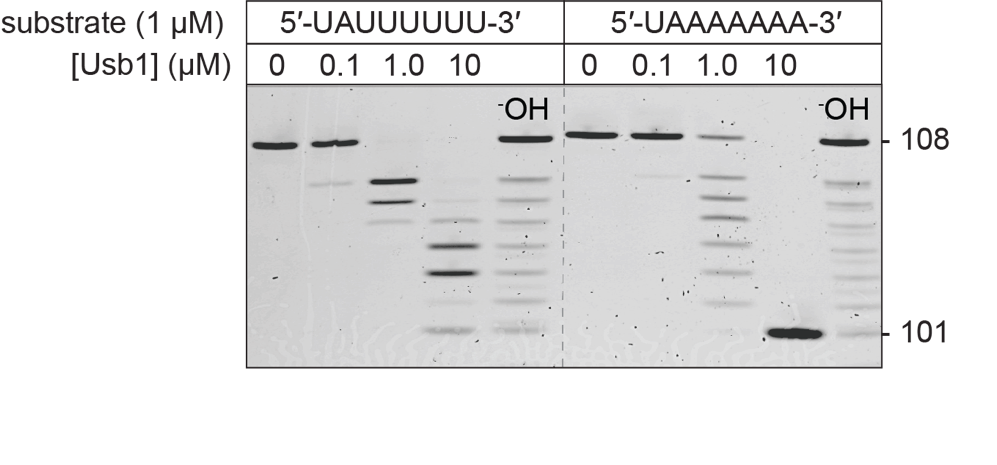


**Supplementary Figure S3.** Comparison of Usb1 processing for oligoA and oligoU RNA substrates.


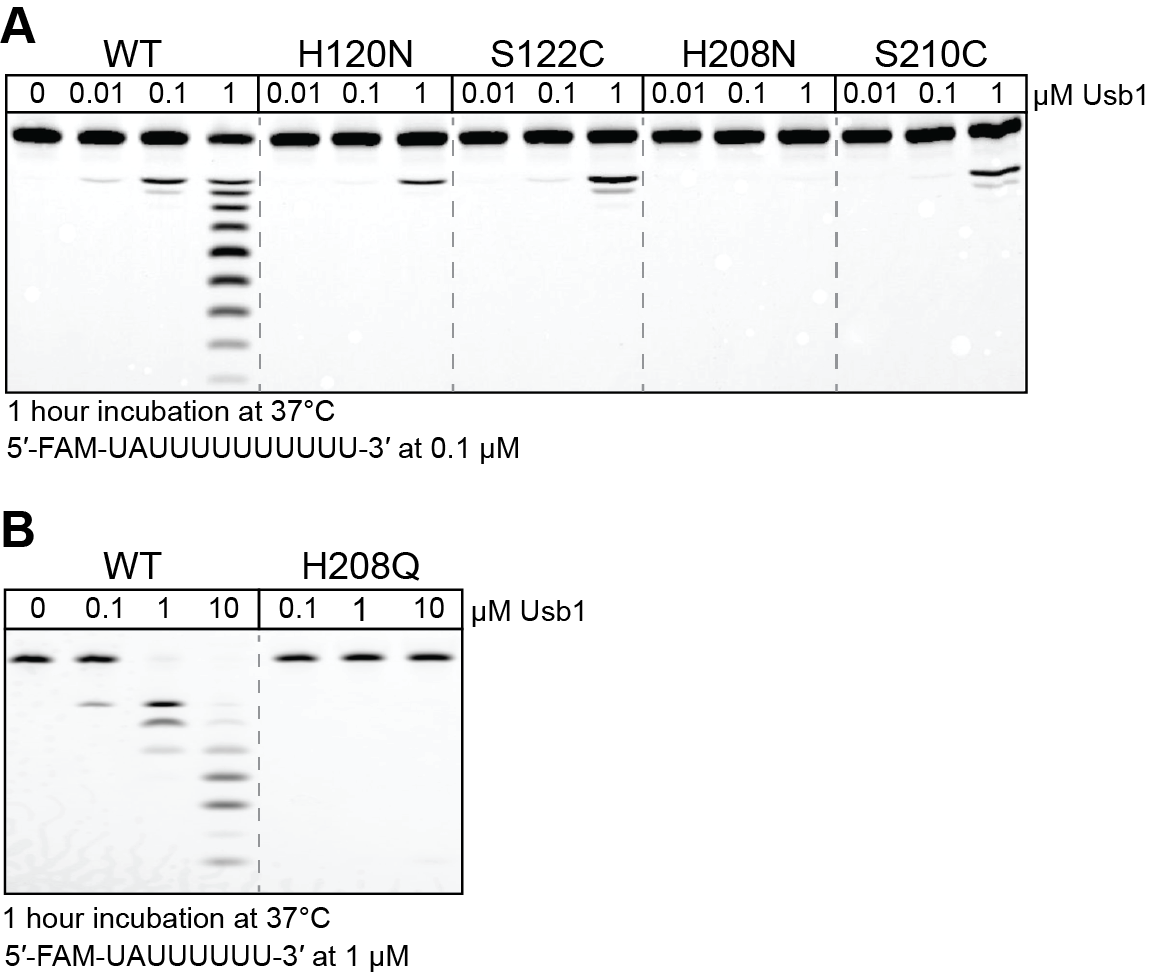


**Supplementary Figure S4.** Mutations in the Usb1 active site inhibit activity. (**A**) Activity comparison of WT and various Usb1 mutants. Concentrations of Usb1 were varied as indicated and incubated with 0.1 µM substrate for 1 hour at 37°C. (**B**) Comparison of WT vs. H208Q. Concentrations of Usb1 were varied as indicated and incubated with 1 µM substrate for 1 hour at 37°C.


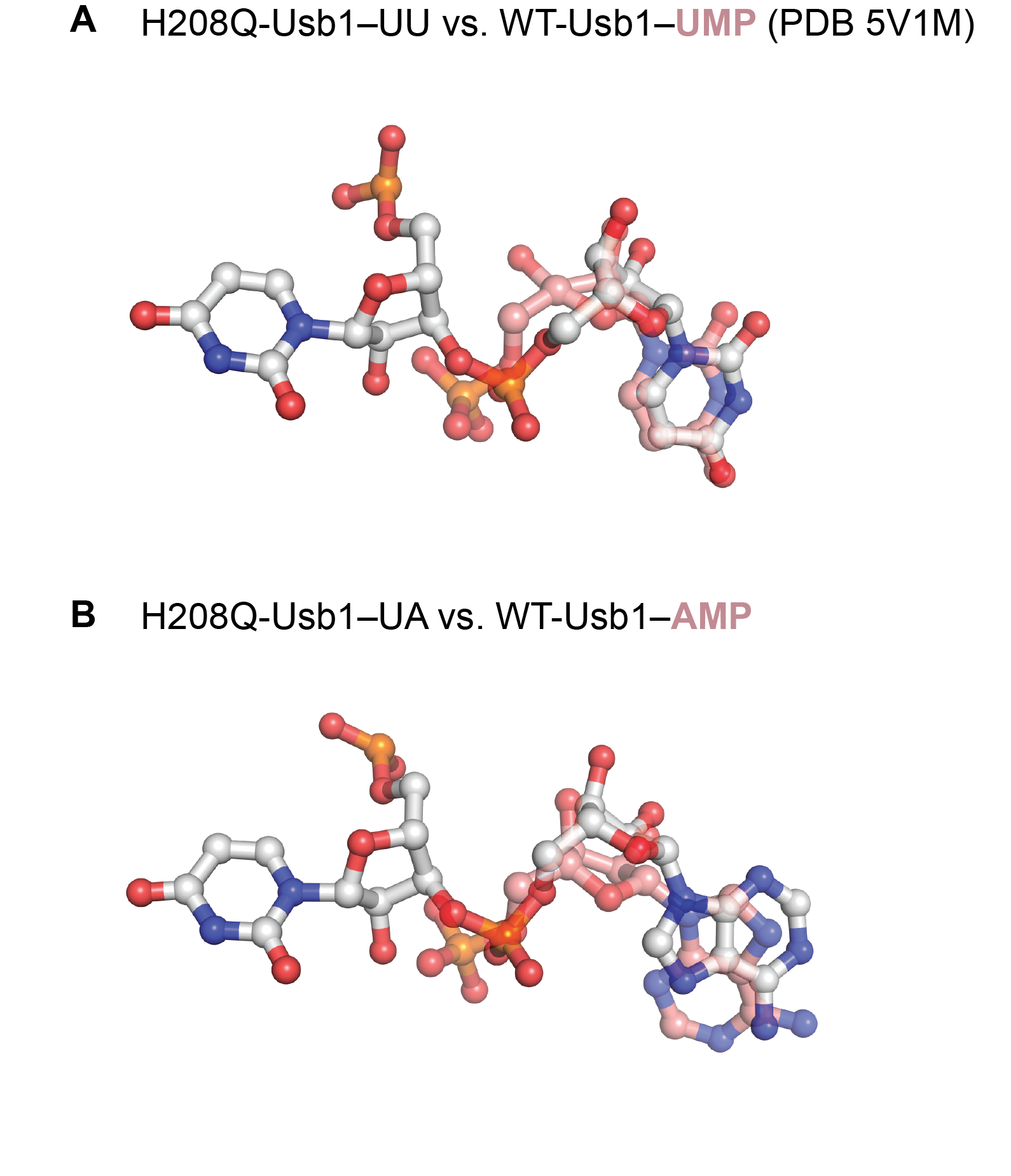


**Supplementary Figure S5.** Displacement of phosphate group by mutation of His208 to glutamine. (**A** and **B**) The phosphate groups in the H208Q-Usb1 structures are displaced by 1 Å relative to the 5ʹ-phosphate groups of UMP and AMP in the WT-Usb1 structures. (**A**) The sugar ring of the terminal uridine forms the C2ʹ-end*o* and C3ʹ-endo puckers in the H208Q and WT structures, respectively. (**B**) The terminal adenosine in the H208Q structure adopts an *anti* configuration and a C2ʹ-endo sugar pucker. The terminal adenosine in the WT structure forms a *syn* configuration and a O4ʹ-endo sugar pucker.

**
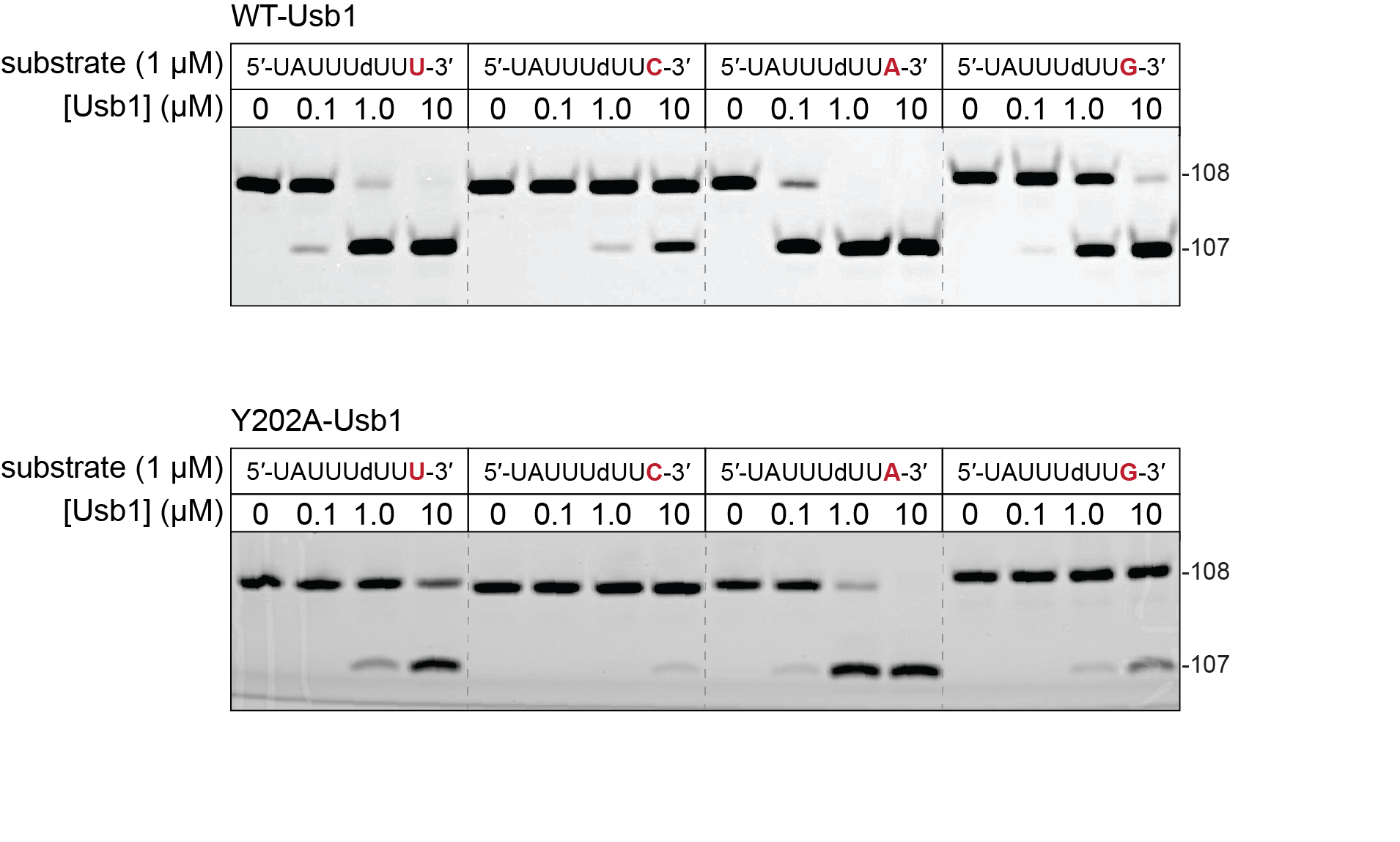
**

**Supplementary Figure S6.** Effect of mutating Tyr202 to alanine on Usb1 catalysis.

**
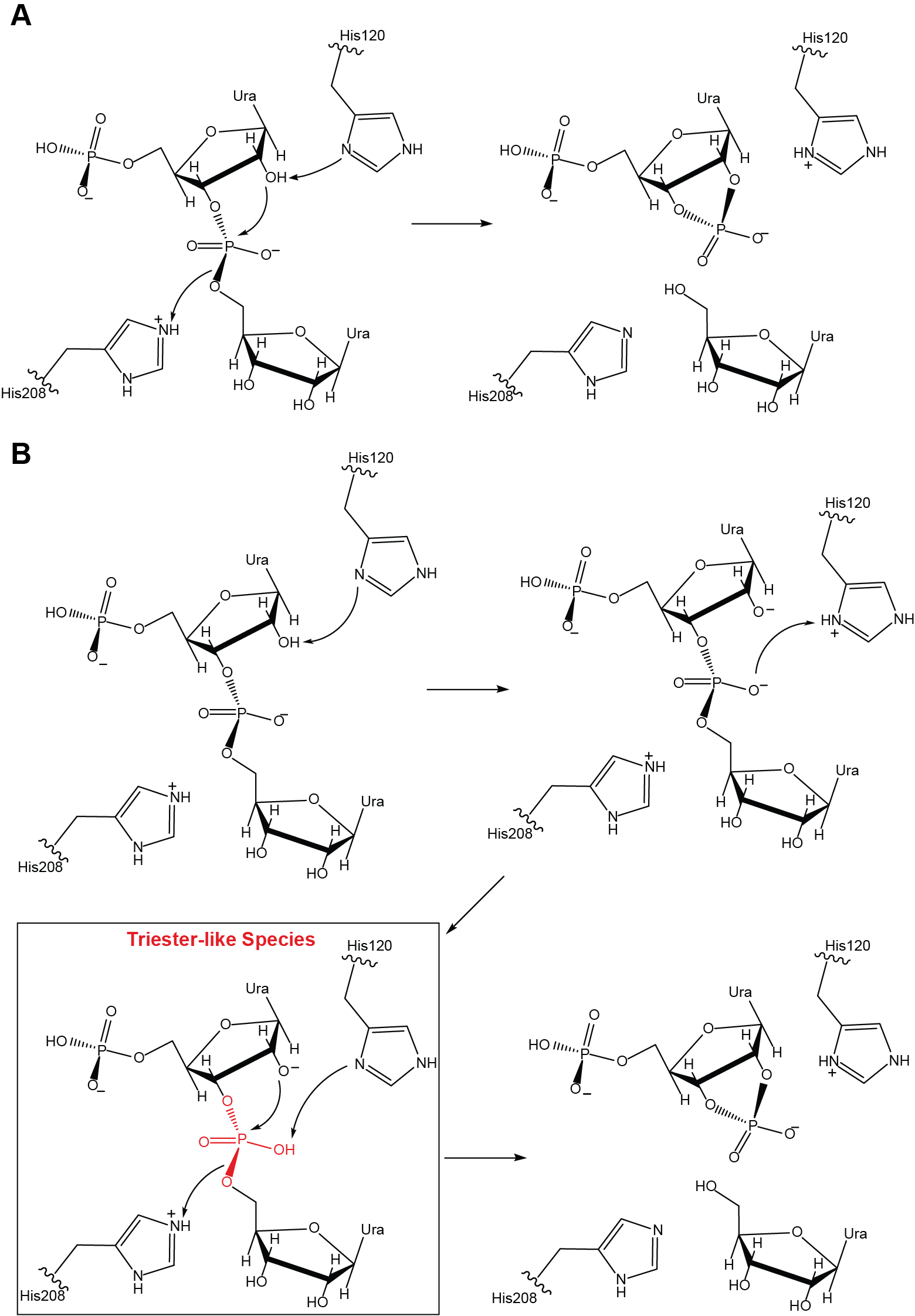
**

**Supplementary Figure S7.** The mechanistic hypotheses tested by simulation and experiment. (**A**) The “classic” mechanism. (**B**) The “triester" mechanism (11). We note that in testing these, the simulations do not make any *a priori* assumptions about whether any processes are concerted or stepwise or even, in fact, whether the species shown are stationary points (e.g. intermediates or transition states) on the FES. The choice to show multiple steps in part B is merely to emphasize the transfer of the proton to and from His120 at multiple points as the reaction progresses and to highlight the presence of the triester-like species.

**
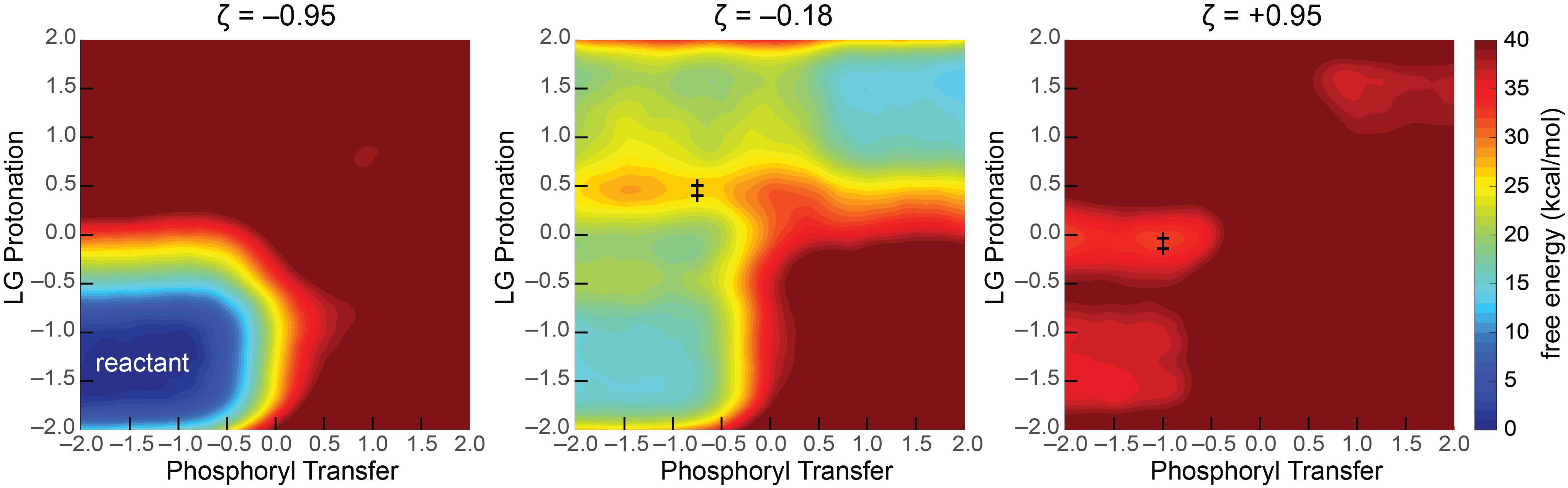
**

**Supplementary Figure S8.** 2D slices of the 3D free energy surface calculated using the ζ coordinate (2) to model the position of the 2ʹ-OH proton. Free energy is in kcal/mol. ζ = –0.95 corresponds to reactant, where the proton is bound to the 2ʹ-O; ζ = +0.95 corresponds to the triester-like species where the proton is bound to the non-bridging oxygen; ζ = –0.18 is a region where the proton is bound neither to donor nor to ultimate acceptor. The stationary point on the ζ = +0.95 surface (indicated by ‡) is significantly higher in free energy than that on the ζ = –0.18 surface. Since it is unlikely to play an important role in this reaction we did not determine whether the stationary point with the triester-like species is an intermediate or a TS. Notably, the stationary point on the ζ = –0.18 surface (‡) is identical to the saddle point located for the classic mechanism in Figure 5. Note that the surfaces here use orthogonal coordinates to those in Figure 5. That is, the slice at ζ = –0.95 would correspond roughly to a slice from the previous surface at “Nuc Deprotonation” = –1.5 (i.e., prior to proton transfer), using the other two coordinates as the axes. Similarly, the slice at ζ = –0.18 would correspond roughly to a slice from the previous surface a “Nuc Deprotonation” = +1.0, where the proton has been transferred to His120. The high free energy at the stationary point at ζ = +0.95 relative to that at ζ = –0.18 indicates that the classic mechanism is more likely.


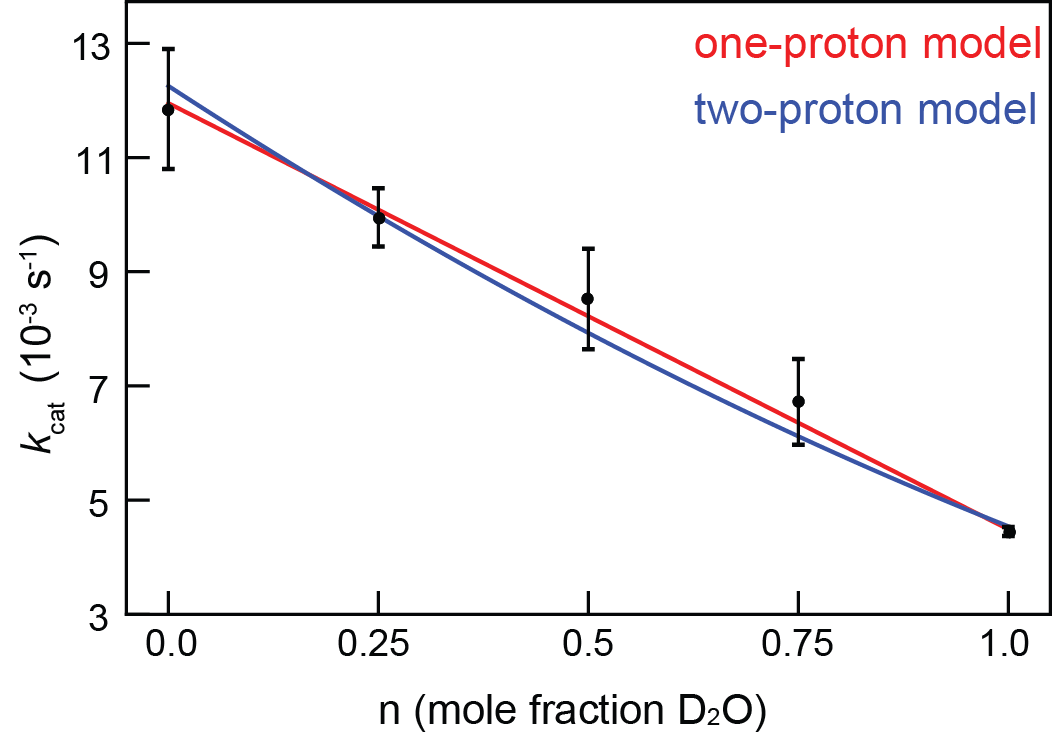


**Supplementary Figure S9.** Proton-inventory for the cleavage reaction of 5′-UAUUUdUUA-3′. The *k*_cat_ was measured with mole fractions of D_2_O to H_2_O designated by “n”. The red and blue lines are fits to models for one-proton catalysis (*k*_n_ = *k*_0_(1 – n + n/[*k*_H_/*k*_D_]) and two-proton catalysis (*k*_n_ = *k*_0_(1 – n + n/[*k*_H_/*k*_D_]_1_) (1 – n + n/[*k*_H_/*k*_D_]_2_)) (12) in the rate-limiting step, respectively. The data fit well to the linear, one-proton model (*r*^2^ = 0.9995) indicating that a model with an additional proton is over-parameterized and unjustified. The measured solvent isotope effect is approximately 2.7, which is within the expected range of around 2–4 (12) for a single proton. Therefore, these data confirm the mechanism and TS predicted by the QM/MM simulations.


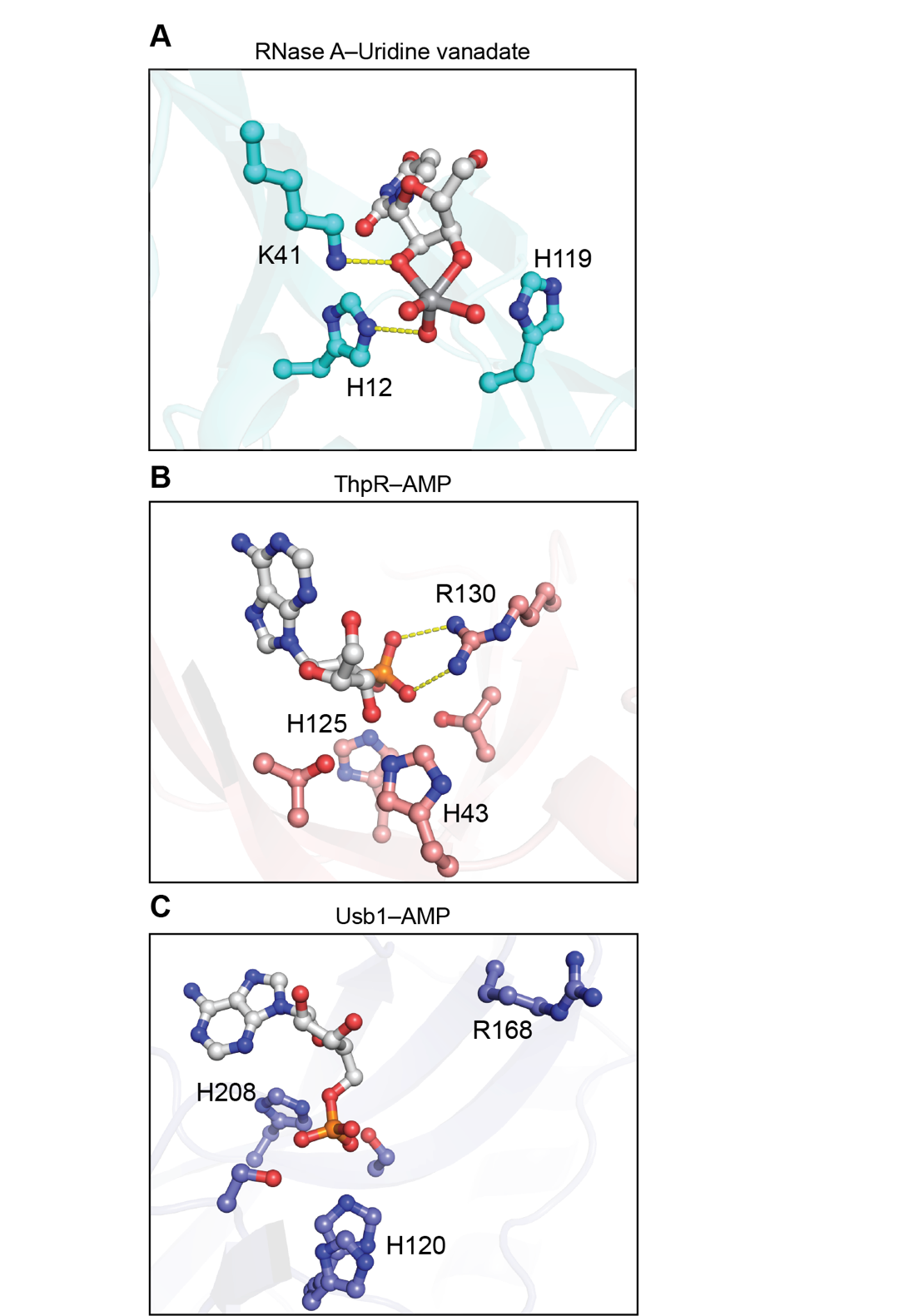


**Supplementary Figure S10.** Charge neutralization in 2H enzyme active sites. (**A** and **B**) Vicinal positive charge likely stabilizes the enzymatic transition state in RNase A and ThpR (PDB ID 1RUV and 4QAK, respectively) (13,14). (**C**) The corresponding residue in human Usb1 is not in close proximity to the labile phosphodiester, as proposed elsewhere (15), thereby providing one rationale for why the enzyme has lower catalytic efficiency relative to other 2H enzymes.


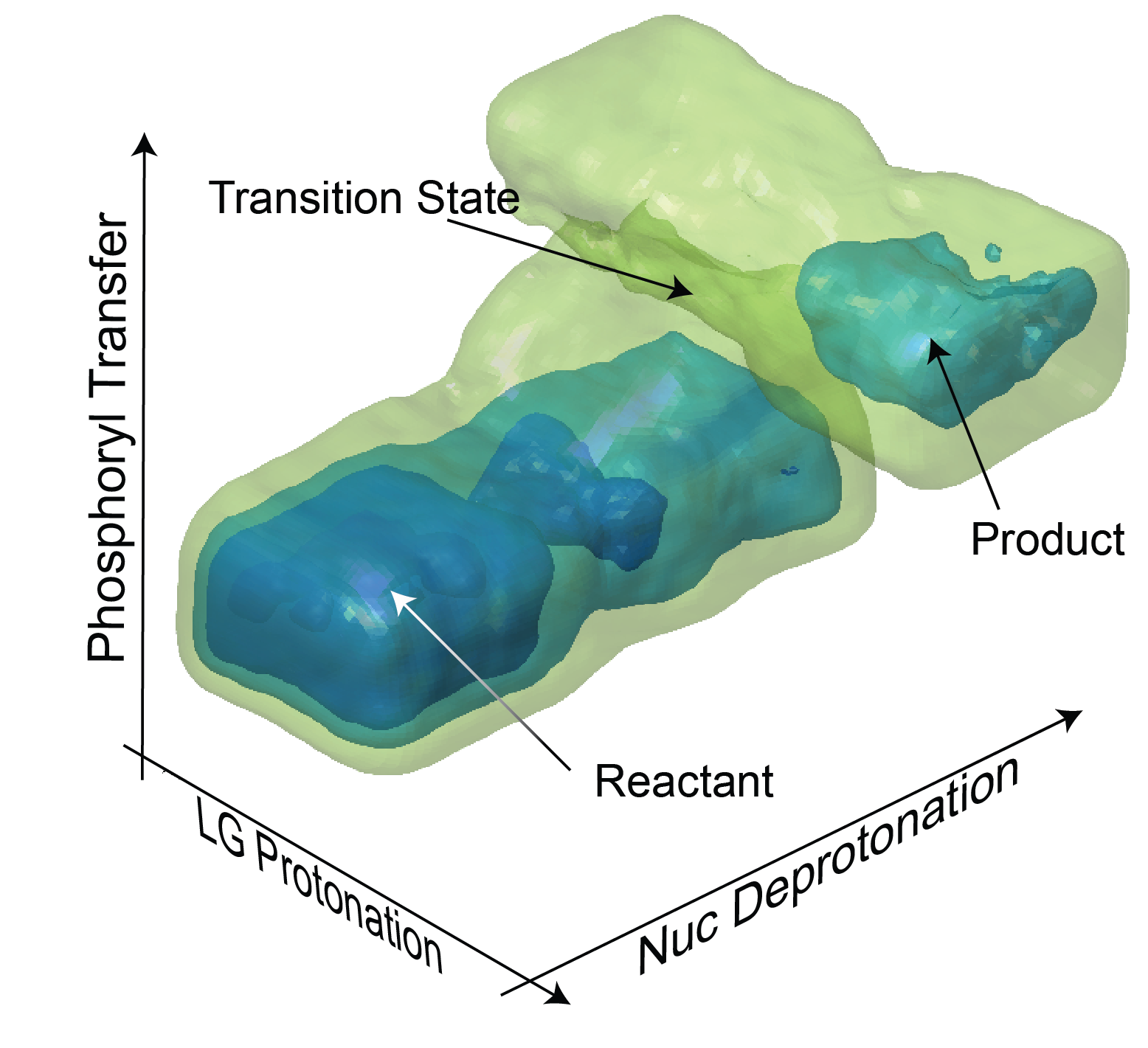


**Supplementary Figure S11.** The full 3D free energy surface that was used to generate the 2D slices in Figure 5B of the main text.

**Supplementary Movie S1.** QM/MM simulation of the cleavage reaction of the UU substrate by Usb1

**Supplementary Movie S2.** QM/MM simulation of the cleavage reaction of the UA substrate by Usb1.

REFERENCES

1. Lad, C., Williams, N.H. and Wolfenden, R. (2003) The rate of hydrolysis of phosphomonoester dianions and the exceptional catalytic proficiencies of protein and inositol phosphatases. *Proc. Natl. Acad. Sci. U. S. A.*, **100**, 5607-5610.

2. Konig, P.H., Ghosh, N., Hoffmann, M., Elstner, M., Tajkhorshid, E., Frauenheim, T. and Cui, Q. (2006) Toward theoretical analyis of long-range proton transfer kinetics in biomolecular pumps. *Journal of Physical Chemistry A*, **110**, 548-563.

3. Wolfram Research, I. (2018). Wolfram Research, Inc., Champaign, Illinois, Vol. Version 11.3.

4. Krezel, A. and Bal, W. (2004) A formula for correlating pKa values determined in D2O and H2O. *Journal of inorganic biochemistry*, **98**, 161-166.

5. Jo, S., Kim, T., Iyer, V.G. and Im, W. (2008) CHARMM-GUI: a web-based graphical user interface for CHARMM. *Journal of computational chemistry*, **29**, 1859-1865.

6. Roston, D. and Cui, Q. (2016) QM/MM Analysis of Transition States and Transition State Analogues in Metalloenzymes. *Methods in enzymology*, **577**, 213-250.

7. Roston, D. and Cui, Q. (2016) Substrate and Transition State Binding in Alkaline Phosphatase Analyzed by Computation of Oxygen Isotope Effects. *J Am Chem Soc*, **138**, 11946-11957.

8. Roston, D., Demapan, D. and Cui, Q. (2016) Leaving Group Ability Observably Affects Transition State Structure in a Single Enzyme Active Site. *J Am Chem Soc*, **138**, 7386-7394.

9. Lu, X., Ovchinnikov, V., Demapan, D., Roston, D. and Cui, Q. (2017) Regulation and Plasticity of Catalysis in Enzymes: Insights from Analysis of Mechanochemical Coupling in Myosin. *Biochemistry*, **56**, 1482-1497.

10. Major, D.T. and Gao, J.L. (2007) An integrated path integral and free-energy perturbation-umbrella sampling method for computing kinetic isotope effects of chemical reactions in solution and in enzymes. *Journal of chemical theory and computation*, **3**, 949-960.

11. Elsasser, B., Fels, G. and Weare, J.H. (2014) QM/MM simulation (B3LYP) of the RNase A cleavage-transesterification reaction supports a triester A(N) + D(N) associative mechanism with an O2' H internal proton transfer. *J Am Chem Soc*, **136**, 927-936.

12. Schowen, R.L. (2007) The use of solvent isotope effects in the pursuit of enzyme mechanisms. *J Labelled Compd Rad*, **50**, 1052-1062.

13. Ladner, J.E., Wladkowski, B.D., Svensson, L.A., Sjolin, L. and Gilliland, G.L. (1997) X-ray structure of a ribonuclease A-uridine vanadate complex at 1.3 A resolution. *Acta crystallographica. Section D, Biological crystallography*, **53**, 290-301.

14. Remus, B.S., Jacewicz, A. and Shuman, S. (2014) Structure and mechanism of E. coli RNA 2',3'-cyclic phosphodiesterase. *RNA*, **20**, 1697-1705.

15. Hilcenko, C., Simpson, P.J., Finch, A.J., Bowler, F.R., Churcher, M.J., Jin, L., Packman, L.C., Shlien, A., Campbell, P., Kirwan, M. *et al.* (2013) Aberrant 3' oligoadenylation of spliceosomal U6 small nuclear RNA in poikiloderma with neutropenia. *Blood*, **121**, 1028-1038.

**Supplementary Table S1.** Geometries during simulations restrained to the TS^a^

|  | UU (Classic)^b^ | UU (Triester)^b^ | UA (*syn*)^c^ | UA (*anti*)^c^ |
| --- | --- | --- | --- | --- |
| *Distance* |  |  |  |  |
| O2'-P | 1.76 ± 0.05 | 1.76 ± 0.05 | 1.77 ± 0.05 | 1.77 ± 0.06 |
| O5'-P | 2.27 ± 0.08 | 2.24 ± 0.08 | 2.28 ± 0.09 | 2.29 ± 0.10 |
| O5'-HE2 (H_208_) | 1.77 ± 0.07 | 1.76 ± 0.10 | 1.76 ± 0.06 | 1.77 ± 0.06 |
| NE2-HE2 (H_208_) | 1.06 ± 0.06 | 1.07 ± 0.09 | 1.06 ± 0.05 | 1.06 ± 0.04 |
| O2'-H2' | 2.09 ± 0.07 | 3.46 ± 0.65 | 2.07 ± 0.06 | 2.09 ± 0.06 |
| H2'-NE2 (H_120_) | 1.03 ± 0.05 | 1.04 ± 0.04 | 1.02 ± 0.03 | 1.03 ± 0.04 |
| O1P-OG (S_122_) | 3.8 ± 0.7 | 4.2 ± 0.6 | 4.0 ± 0.4 | 3.5 ± 0.6 |
| O2P-OG (S_210_) | 3.7 ± 1.1 | 6.1 ± 1.0 | 2.9 ± 0.4 | 4.7 ± 1.0 |
| *Angle* |  |  |  |  |
| O2'-P-O5' | 170 ± 4 | 170 ± 4 | 166 ± 4 | 169 ± 5 |
| O5'-HE2-NE2 (H_208_) | 163 ± 9 | 164 ± 9 | 154 ± 10 | 165 ± 8 |
| O2'-H2'-NE2 (H_120_) | 138 ± 19 | 135 ± 23 | 147 ± 15 | 139 ± 16 |

^a^ The position of the TS was determined from the FESs of UU in Figure 5 and Supplementary Figure S8. The two UA simulations assumed that in terms of the 3 primary reaction coordinates the position of the TS was similar for the two substrates.

^b^ As the geometries indicate, the two UU simulations represent the same state, i.e., the classic TS. The column labeled “Triester” is the stationary point found at ζ = –0.18 in the simulations testing the triester mechanism and the TS was sampled by restraining ζ, not by restraining the O2ʹ-H2ʹ-NE2 antisymmetric stretch; hence the increased flexibility in the O2ʹ-H2ʹ distance in the triester simulations.

^c^ The UA(*syn*) simulation has a starting structure with the adenosine in the *syn* conformation while UA(*anti*) has the adenosine in the *anti* conformation. This difference explains some of the variation observed in TS geometries. Note that during the simulations there were no restraints on the conformation of the adenosine.
